# Characteristics of Lower Extremity Kinematics, Kinetics, and Muscle Activity in Individuals With Chronic Ankle Instability During Landing With Expected and Unexpected Inversion Perturbations: A Systematic Review and Meta‐Analysis

**DOI:** 10.1002/jfa2.70082

**Published:** 2025-09-08

**Authors:** Yixue Gong, Menghan Xu, Peng Chen, Xiaomei Hu, Wenxing Zhou, Lin Wang

**Affiliations:** ^1^ School of Exercise and Health Shanghai University of Sport Shanghai China; ^2^ Department of Sports Science and Physical Education The Chinese University of Hong Kong Hong Kong China; ^3^ Sports Medicine and Rehabilitation Center Shanghai University of Sport Shanghai China

**Keywords:** biomechanics, chronic ankle instability, meta‐analysis, postural control

## Abstract

**Objective:**

This study aims to investigate whether alterations in the lower extremity kinematics, kinetics, and muscle activity of individuals with chronic ankle instability (CAI) occur during landing with expected and unexpected inversion perturbations.

**Methodology:**

PubMed, Embase, Cochrane Library, Web of Science, and Scopus databases were searched for relevant studies up to November 30, 2024. Comparative studies investigating the characteristics of lower extremity kinematics, kinetics, and muscle activity in individuals with CAI compared with healthy controls were included. Two independent reviewers extracted the data. Certainty of the evidence was assessed using the Newcastle–Ottawa Scale (NOS) approach.

**Synthesis:**

Thirteen studies involving 207 patients with CAI and 215 healthy controls were included. Individuals with CAI exhibited increased activity of the tibialis anterior muscle before landing (SMD = 0.28 and 95% CI: 0.03–0.54). The delayed activation of the peroneus longus muscle (SMD = 1.35 and 95% CI: 0.90–1.80) and increased co‐contraction index in the sagittal plane (SMD = 0.41 and 95% CI: 0.06–0.77), ankle inversion angle (SMD = 0.56 and 95% CI: 0.30–0.81), ankle inversion range of motion (SMD = 0.83 and 95% CI: 0.42–1.24), and knee extension moment (SMD = 0.71 and 95% CI: 0.32–1.11) were observed after landing. Besides, subgroup analysis revealed that the anticipation of perturbations influenced muscle activation patterns, with significant differences in peroneus longus latency and coactivation indices.

**Conclusion:**

Patients with CAI may present differences in lower extremity biomechanics during expected and unexpected inversion‐perturbed landings compared with healthy controls. The results of this work may have clinical implications in the development of more effective and targeted rehabilitation programs for individuals with CAI.

**Trial Registration:**

PROSPERO registration number: CRD42024615006.

## 1 Introduction

Chronic ankle instability (CAI) is a common issue in musculoskeletal injuries [1, 2, 3]. A total of 32%–74% individuals with CAI report recurrent lateral ankle sprain (LAS), persistent pain, and the feeling of ankle instability [4]. The development of CAI further leads to the damage of the anterior cruciate ligament [5], exercise fear, and early‐onset osteoarthritis [6]. Furthermore, managing CAI imposes significant healthcare costs [3, 4, 7]. Despite various proposed treatments, their effectiveness is limited due to the complex mechanisms underlying CAI. Investigating the contributory factors and associated functional limitations of CAI is essential to develop related interventions and management approaches [8].

Landing, as a risk factor for LAS [9], has been widely investigated [8, 10, 11]. Such movement may increase ankle injury risk and affect functional performance, particularly in sports and competition activities [12, 13], in patients with CAI. The deafferentation of joint mechanoreceptors due to ankle sprains could chronically suppress gamma activation and desensitize muscle spindles [14], thereby resulting in inaccurate proprioceptive feedback to the central nervous system (CNS) and abnormal motor behaviors [15]. Consequently, individuals with CAI may struggle to adjust their ankle movements promptly during landing, especially under perturbation conditions, leading to further LAS [10, 13, 16, 17].

Recent studies have investigated motor performance during landing with an inversion disturbance (LID), which is suggested to simulate LAS [18]. Inversion perturbation during landing has been observed to alter motor control of patients with CAI, including their ankle muscle contraction [19, 20] and kinematics, further adversely influencing knee biomechanics [21]. However, some studies have observed that individuals with CAI may display similar motor control and modulation strategies during inversion landing [19, 22, 23, 24, 25, 26]. They may rapidly adjust motor control strategies through anticipatory postural adjustments to compensate for sensorimotor dysfunction during expected conditions [23, 27, 28, 29, 30]. Their motor control pattern can be classified into feedforward and feedback strategies [31]. Anticipatory postural adjustments often play a crucial role in feedforward strategies [32]. By contrast, feedback strategies involve compensatory postural adjustments that invoke reactive muscle activation, helping patients with CAI maintain postural stability under unexpected conditions [22, 28, 33, 34, 35].

In the present context, the unexpected condition refers to landing tasks in which participants are prevented from knowing the upcoming platform condition—whether flat or inverted—before landing. This is typically achieved by methods, such as having participants face away from the landing platform, while the inversion setting is changed or requiring them to look straight ahead during the landing task. Conversely, in the expected condition, participants are aware of the platform condition prior to execution [19, 20, 22, 23, 28, 34, 35].

However, consensus on the strategies used by CAI has not been reached. Some researchers have found reduced tibialis anterior (TA) muscle activity and frontal co‐contraction index (fCCI) after landing in CAI compared with healthy control [34], whereas others have observed increased fCCI [23]. Besides, Lee et al. [22] reported increased TA in CAI before and after landing. The motor patterns of CAI during inversion landing remain unclear.

Despite these findings, there is no systematic review that directly compares how individuals with CAI and healthy controls employ motor control strategies under both expected and unexpected inversion perturbation conditions—tasks that closely resemble real‐world ankle injury scenarios. To our knowledge, this is the first systematic review to categorically synthesize the evidence on these strategies, with the goal of clarifying potential differences and informing both injury mechanism research and the development of more effective clinical assessment tools and targeted rehabilitation strategies.

## 2 Method

This systematic review was conducted in accordance with the guidelines provided by the Preferred Reporting of Systematic Reviews and Meta‐Analysis (PRISMA) statement [36] (PROSPERO registration number CRD42024615006).

### 2.1 Search Strategy

Articles were searched in PubMed, Embase, Cochrane Library, Web of Science, and Scopus. The specific search strategy can be found in Supporting Information S1: Supporting Information 1 Table S1. All the publications were searched until November 30, 2024, without any restrictions. The reference lists of included articles were screened to identify additional studies.

### 2.2 Selection Criteria

Search results were independently screened in accordance with predetermined inclusion and exclusion criteria by two reviewers using EndNote X9 (Clarivate Analytics, British), with adjudication by a third author if necessary. The inclusion criteria were (1) comparative studies that presented data for participants with CAI and a unique group of healthy controls (not just comparing injured vs. uninjured sides of those with CAI) during a landing task on an unexpected/or expected tilted/or flat surface. We identified the landing with inversion disturbance caused by an inverted landing surface as an LID task; (2) reported outcomes on lower limb kinematics, kinetics, or electromyograph (EMG); (3) CAI and controls were screened by using validated ankle instability questionnaires [37]; and (4) published in a peer‐review journal. Studies that did not use the term CAI and analyzed groups with functional ankle instability instead were included as long as the aforementioned criteria were met because the consensus statement for the consistent terminology of CAI has only recently been published [37]. The exclusion criteria were (1) task using lateral or diagonal directions; (2) interventional studies without reported outcomes prior to the intervention; (3) case studies, case series, guidelines, and reviews; and (4) literature with less than five Newcastle–Ottawa Scale (NOS) stars.

### 2.3 Methodological Quality Assessment

Study quality and risk of bias was assessed by using the NOS tool. Although originally developed for epidemiological case–control and cohort studies, the NOS has been adopted in biomechanics reviews for similar CAI versus control comparisons [5, 38, 39]. A “star system” has been developed for NOS [40, 41]. In this system, a study is judged from three broad perspectives: the selection of study groups, comparability of groups, and ascertainment of either the exposure or outcome of interest for case–control or cohort studies. Studies given an overall scoring of less than five stars were considered to have low quality and excluded [38, 39]. The assessment was conducted by two reviewers. Wherein the scores for a given study did not align with each other, a senior author independently reviewed the article and decided the outcome. The specific evaluation standard is presented in Supporting Information S1: Supporting Information 2.

### 2.4 Data Extraction

A Microsoft Excel (2021) table was used to extract data from the included studies pertaining to the study design, sample size, information on population demographics, participant inclusion/exclusion criteria, landing protocol, primary outcome measures, and significant findings. A third researcher was consulted if any issue could not be resolved.

All reported outcomes were divided into two phases in accordance with initial contact (IC), either prelanding (before IC) or postlanding (after IC). Studies reporting expected and unexpected conditions were treated as two independent data sets to include as much data as possible, this classification method of independent variable data has also been used before [42, 43, 44]. When at least two studies reported the same primary outcome during the same phase, their findings were analyzed as continuous data and combined in a meta‐analysis. This approach was subject to the adequate reporting of means, SD, or effect sizes. Otherwise, qualitative synthesis was conducted. When data were not reported, we estimated effect sizes on the basis of reported *p* values and/or 95% confidence intervals (CIs) by using the methods described by previous studies [45, 46]. Data that were only presented in the form of images were extracted by using GetData Graph Digitizer (version 2.25). Parameters without an exact value were reported as missing data.

### 2.5 Data Analysis

Our primary outcomes were the joint angle, range of motion (ROM), moment, and muscle activation of lower extremity joints before and/or after landing. The statistical analysis of pooled data was performed by using Review Manager (RevMan, version 5.3). Standardized mean differences (SMDs) between CAI and healthy controls were described by using Cohen's *d*. Effect sizes were considered small when 0.2 ≤ SMD < 0.5, medium when 0.5 ≤ SMD < 0.8, and large when SMD ≥ 0.8 [47]. Given that we anticipated considerable between‐study heterogeneity, we used a random‐effects model to compute the pooled SMDs and corresponding 95% CIs to examine muscle activation in CAI and control groups. Conversely, given their kinematic and kinetic characteristics, a fixed‐effects model was utilized to consolidate the estimation of effect sizes initially. Kinematic results were also analyzed and reported as mean differences (MDs) for clinical applicability [48]. Data were analyzed by comparing the more injured limb of the CAI group with the dominant limb of the control group.

Heterogeneity between studies was evaluated by using the *I* ^2^ and Q statistics. The *I* ^2^ index was employed to assess statistical heterogeneity between pooled results, and its values were interpreted as low ≤ 50%, moderate = 51%–74%, and large ≥ 75% [49]. In the presence of considerable between‐study heterogeneity (*I* ^2^ > 50%) [50], further analyses, including sensitivity and subgroup analyses, would be performed to explore possible sources of heterogeneity and improve the robustness of meta‐analyses. First, a fixed‐effects model was used to analyze indices of *I* ^2^ ≤ 50%, and a random‐effects model was employed for those of *I* ^2^ > 50%. Second, for the parameters of *I* ^2^ > 50%, we used the leave‐one‐out method in RevMan to identify influential cases when > 3 studies were present in each pooled analysis. The influential study was omitted, and the pooled effect was reinspected. Subgroup analyses were conducted when applicable. Predefined subgroups included the anticipation (unexpected and expected) of disturbance during landing. Forest plots were presented for the meta‐analysis of SMD. Statistical significance was set at *p* < 0.05.

### 2.6 Risk of Publication Bias

After reviewing the meta‐analysis data through forest plots, the asymmetry of the effect size was first judged visually by using funnel plots. In addition, the relationship between the effect size and standard error was verified by using Egger's regression to determine whether a funnel plot was asymmetric or not. We adjusted the average effect size through the trim‐and‐fill method [51].

## 3 Results

### 3.1 Search Results

**FIGURE 1:**
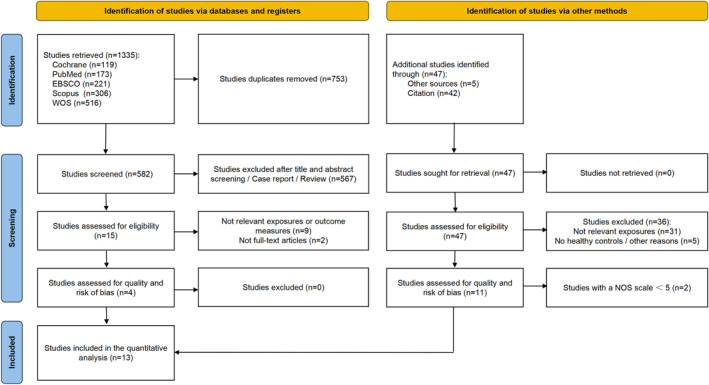
Flowchart of article screening.

The electronic search conducted on November 30, 2024, yielded 1335 potentially relevant articles. After duplicate removal, 753 articles were screened by title and abstract. Fifteen articles were assessed for eligibility in accordance with the inclusion/exclusion criteria and their full texts. An additional 11 records were included through cross‐referencing. Finally, 13 articles [19, 20, 21, 22, 23, 24, 25, 26, 28, 34, 35, 52, 53] met the inclusion criteria and were included in the present review (Figure 1).

### 3.2 Characteristics of the Included Studies

A total of 207 patients with CAI (mean age 23.46 years), as well as 215 controls (mean age 23.13 years), were included in this review. Among the 13 studies, drop landing was the most common type [21, 23, 25, 28, 34, 52] and another one was step‐down [24]. Two works used the bilateral‐landing task with a trapdoor to simulate inversion disturbance [19, 20], two studies applied with a tilt platform [21, 25], whereas most investigations used unilateral‐landing task with a tilt platform [22, 23, 26, 28, 34, 35, 52, 53], and another research conducted unilateral‐landing with a fulcrum [24]. Nine studies reported group differences in landing trials with expected disturbance [20, 21, 24, 25, 26, 34, 35, 52, 53], and seven works compared the between‐group results under unexpected condition [19, 20, 22, 23, 28, 35, 54]. In addition to one research that only utilized a combined angle design [19], the inversion angles of perturbations involved a wide range from 5° to 30°, with 25° being the most frequently used (*n* = 8), and their mean (SD) was 21.67 (8.07)°. The landing heights involved in this study ranged from 15 to 46 cm, with 30 cm (*n* = 9) being the most commonly used, and their mean (SD) was 28.85 (7.07) cm (Table 1).

**TABLE 1:** Basic characteristics of the included literature.

Author/year	Country	Exposure	Sample size	Age (year) ± SD	Angle (°) ± SD	Height (cm)	Anticipation	Frequency	Comparisons	Primary outcomes
Gutierrez 2012	America	Bilateral landing + trapdoor	15 AI/15CON	18–30 (20.9 ± 2.9)	4.5I + 5PF + 8AD	30	Both	22 trials (including two familiarization trials)	Group	EMG and kinematics
Han 2023	America	Unilateral landing + tilt platform	22 CAI (11M11F)/22 Con (11M11F)	23.4 ± 2.4/23.4 ± 2.6	25	30 (gap)	Both	Familiarization trial, five EXP and 10 UE trials, 60 s intervals	Anticipation Group	Kinematics
Knight 2011	America	Unilateral landing + fulcrum (outer sole)	15 LAS/15 CON	21.07 ± 1.1/21.2 ± 1.26	25	24	EXP	10 trials	Group	EMG
Lee 2023	America	Unilateral landing + tilt platform	22 CAI (11M11F)/22 CON (11M11F)	23.4 ± 2.4/23.4 ± 2.6	25	30 (gap)	UE	5–10 familiarization trials, seven flat trials, and seven tilted trials	Group	EMG and kinematics
Levin 2015	Belgium	Bilateral landing + trapdoor	9 CAI (4M5F)/9 CON (5M4F)	21.1 ± 1.36/23.7 ± 4.24	25	20 (gap)	Both	40 trials	Anticipation Group Testing limb Disturbance type	EMG
Li 2018a	America	Bilateral drop landing + tilt platform	21 CAI (21F)/21 CON (21F)	21 ± 2/21 ± 2	25	30 (gap)	EXP	10 trials	Group	EMG
Li 2018b	America	Bilateral drop landing + tilt platform	21 CAI (21F)/21 CON (21F)	21 ± 2/21 ± 2	25	30 (gap)	EXP	10 trials	Group	Kinematics and kinetics
Liu 2013	America	Unilateral landing + tilt platform	6 CAI (6M)/11 CON (11M)	24 ± 2.1/24.67 ± 2.42	25	30 (gap)	EXP	Five trials under four drop landing conditions	Group Disturbance type	Kinematics and kinetics
Moisan 2020	Canada	Unilateral drop landing + tilt platform	32 CAI/31 CON	18–45	25	46 (gap)	EXP	Five trials of SIDE/DROP/FOAM/WEDGE task	Group Disturbance type	EMG, kinematics, and kinetics
Simpson 2019	America	Unilateral drop landing + tilt force plate	15 CAI/15 CON	21.3 ± 1.6/21.5 ± 1.5	20	30 (gap)	Both	Up to 10 flat trials with a sudden UE inversion trial and an EXP inversion trial, 60 s intervals	Anticipation Group	EMG and kinematics
Song 2018	China	Unilateral landing + flat force plate	11 FAI (11F)/11 CON (11F)	22.85 ± 2.15/23.06 ± 3.05	30	15	EXP	Familiarization trial, five continuous formal trials, foot rotation after 60 s intervals	Group Contralateral limb	EMG and kinematics
Watabe 2021a	Japan	Unilateral drop landing + tilt platform	8 CAI/8 CON	20.1 ± 1.2/19.7 ± 1.3	0/5 (I/E) at IC	30	UE	5–10 familiarization trials, instruction displayed on a laptop, three successful trials in the right/left/forward direction	Group Disturbance type	EMG
Watabe 2021b	Japan	Unilateral drop landing + tilt platform	12 CAI (12M)/12 CON (12M)	20.1 ± 1.2/19.7 ± 1.3	0/5 (I) at IC	30	UE	5–10 familiarization trials, 10 trials (three tilted and seven flat trials)	Group Disturbance type	EMG and kinematics

Abbreviations: AD = adduction, CAI = chronic ankle instability group, Con = control group, DROP = drop landing, E = eversion, EMG = electromyogram, F = female, FAI = functional ankle instability group, FOAM = drop landing on an unstable surface, I = inversion, IC = initial contact, L = left, LAS = lateral ankle sprains group, M = male, PF = plantarflexion, R = right, SIDE = maximal side jump landing, WEDGE = drop landing on a 25° laterally inclined surface.

Primary outcomes were compared by using a control group in 13 studies [19, 20, 21, 22, 23, 24, 25, 26, 28, 34, 35, 52, 53] and the contralateral or nontested leg in two works [20, 26]. Furthermore, five studies [20, 23, 28, 52, 53] compared primary outcomes under different disturbance types (Supporting Information S1: Supporting Information 3 Table S2–Supporting Information 5 Table S4). A summary of the included studies is presented in Table 1. The specific inclusion criteria for participants are also reported in Supporting Information S1: Supporting Information 6 Table S5.

### 3.3 Assessment of Methodological Quality

All included studies reported their aims/hypotheses, participant characteristics, and primary outcomes and used valid and reliable assessment and outcome tools. The most frequent source of potential bias was the lack of information on nonresponse rates (*n* = 13) and control selection (*n* = 8) (Table 2).

**TABLE 2:** Quality assessment of the included studies.

Study	Selection				Comparability	Exposure			Quality scores
	Adequate definition of cases	Representativeness of cases	Selection of controls	Definition of controls	Control for important factors^a^	Ascertainment of exposure	Same method ascertainment for cases and controls	Nonresponse rate	
Gutierrez 2012	☆	☆	☆	☆	☆☆	☆	☆		8
Han 2023	☆	☆		☆	☆☆	☆	☆		7
Knight 2011	☆	☆		☆	☆☆	☆	☆		7
Lee 2023	☆	☆	☆	☆	☆☆	☆	☆		8
Levin 2015	☆			☆	☆☆	☆	☆		6
Li 2018a	☆	☆		☆	☆☆	☆	☆		7
Li 2018b	☆	☆		☆	☆☆	☆	☆		7
Liu 2013	☆	☆		☆	☆☆	☆	☆		7
Moisan 2020	☆	☆	☆	☆	☆☆	☆	☆		8
Simpson 2019	☆	☆	☆	☆	☆☆	☆	☆		8
Song 2018	☆	☆		☆	☆☆	☆	☆		7
Watabe 2021a	☆	☆	☆	☆	☆☆	☆	☆		8
Watabe 2021b	☆	☆		☆	☆☆	☆	☆		7

^a^ A maximum of two stars can be allotted in this category: one for age and the other for other controlled factors.

### 3.4 Quantitative Synthesis: Meta‐Analysis

#### 3.4.1 Electromyography

**FIGURE 2:**
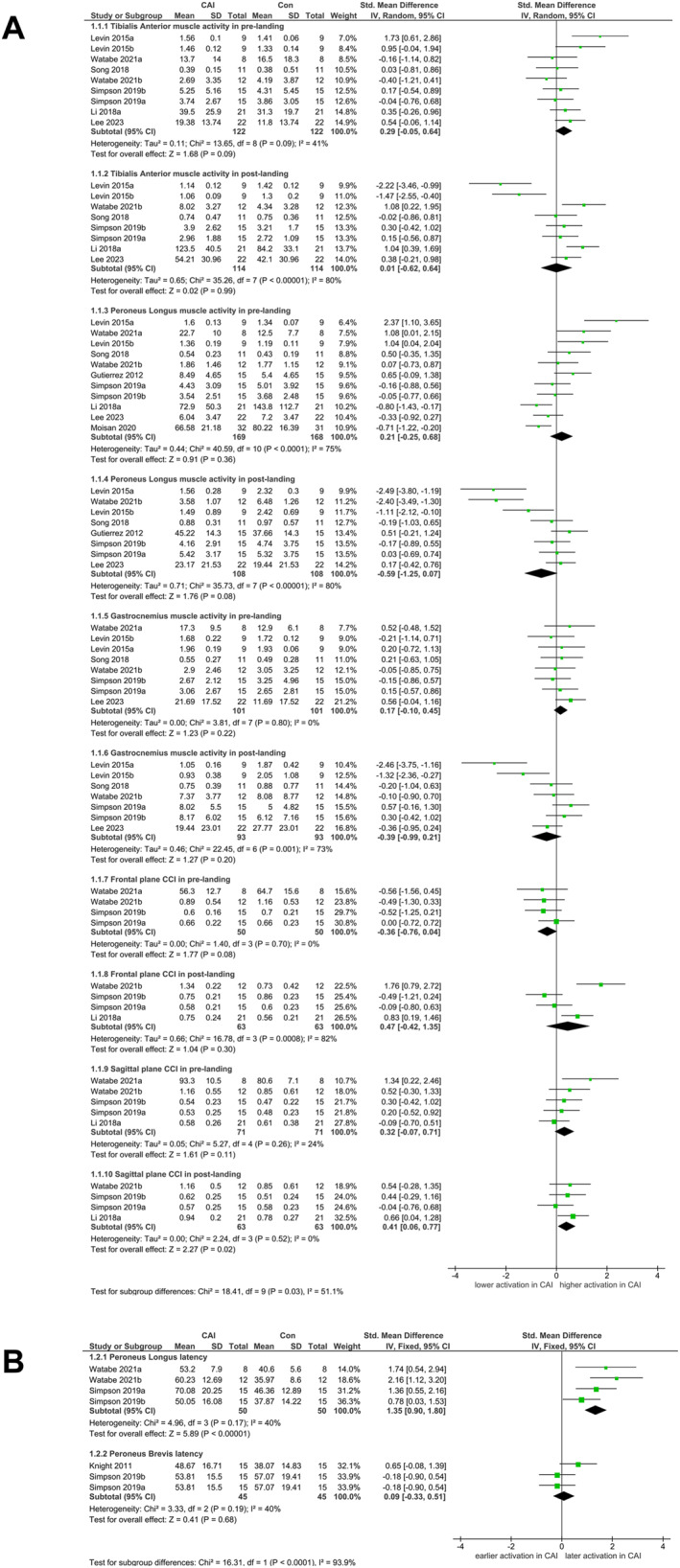
Forest plot of pre‐LID and post‐LID electromyogram characteristics comparing CAI with control. (A) Pooled estimates for muscle activity using a random‐effects model and (B) pooled estimates for latency using a fixed‐effects model. CAI = chronic ankle instability group; Con = healthy control group; IV = inverse variance.

Pooled analysis revealed a small effect regarding the significantly higher TA activity amplitude before landing in patients with CAI than in healthy controls (SMD = 0.28, 95% CI [0.03, 0.54], *p* = 0.03, and Figure 2A; Supporting Information S1: Supporting Information 7 Table S6). No clear asymmetries were identified in the funnel plots for the related parameters except for peroneus longus (PL) muscle activity (Supporting Information S1: Supporting Information 8 Figure S1). No significant changes in pooled SMD were found in sensitivity analyses, indicating that the results of meta‐analysis are reliable.

The frontal plane CCI (fCCI) of muscles surrounding the ankle joint in prelanding phase showed significant changes in overall results after we excluded the study by Li et al. [25] (Supporting Information S1: Supporting Information 9 Table S7). The reinspected pooled effects revealed no significant group differences for fCCI and sagittal plane CCI (sCCI) (*p* > 0.05 and Figure 2A; Supporting Information S1: Supporting Information 7 Table S6). The asymmetry was only identified for sCCI (Supporting Information S1: Supporting Information 8 Figure S1).

In the postlanding phase, the pooled effects revealed no significant group differences (*p* > 0.05 and Figure 2A; Supporting Information S1: Supporting Information 7 Table S6). No clear asymmetries were identified for the related parameters except for the postlanding TA and PL muscle activities (Supporting Information S1: Supporting Information 8 Figure S1). No significant changes were found in sensitivity analyses.

The reinspected pooled effects revealed a small effect regarding significantly increased sCCI in CAI group after landing (SMD = 0.41, 95% CI [0.06, 0.77], *p* = 0.02, and Figure 2A; Supporting Information S1: Supporting Information 7 Table S6). No clear asymmetries were identified (Supporting Information S1: Supporting Information 8 Figure S1). No significant changes in sensitivity analyses were found.

The PL latency showed a significant change after we removed the articles written by Knight and Weimar [24] (Supporting Information S1: Supporting Information 9 Table S7). After reinspection analysis, the pooled data showed a large effect regarding the significantly delayed activation of PL in the CAI group (SMD = 1.35, 95% CI [0.90, 1.80], *p* = 0.03, and Figure 2B; Supporting Information S1: Supporting Information 7 Table S6) but no difference for PB (*p* > 0.05 and Figure 2B; Supporting Information S1: Supporting Information 7 Table S6). No clear asymmetries were identified in the funnel plots (Supporting Information S1: Supporting Information 8 Figure S1).

#### 3.4.2 Kinematics

**FIGURE 3:**
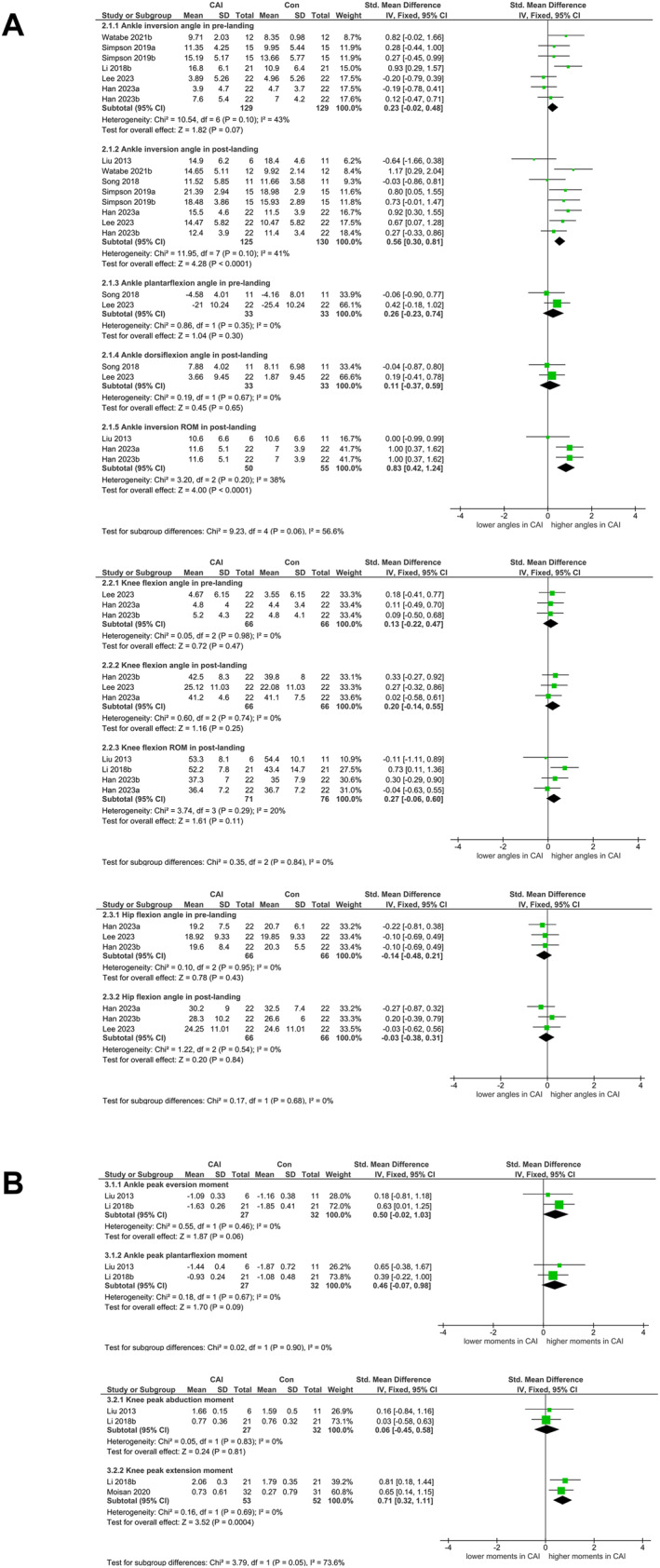
Forest plot of pre‐LID and post‐LID kinematic and kinetic comparing CAI with control. (A) Pooled estimates for kinematics obtained by using fixed effects model and (B) pooled estimates for kinetics using a fixed effects model. CAI = chronic ankle instability group; Con = healthy control group; IV = inverse variance; ROM = range of motion.

No significant group differences were observed in ankle inversion and plantarflexion angles before landing (*p* > 0.05 and Figure 3A; Supporting Information S1: Supporting Information 10 Table S8). No clear asymmetries were identified (Supporting Information S1: Supporting Information 8 Figure S1). The exclusion of individual studies did not trigger a significant change in sensitivity analyses. The overall results for postlanding ankle inversion angle and inversion ROM showed significant changes after we removed the articles written by Gutierrez et al. [19] and Li et al. [21], respectively (Supporting Information S1: Supporting Information 9 Table S7). Therefore, relative to those in the control group, pooled data showed a medium effect for higher inversion angle (SMD = 0.56, 95% CI [0.30, 0.81], *p* < 0.001, and Figure 3A; Supporting Information S1: Supporting Information 10 Table S8) and a large effect for higher inversion ROM (SMD = 0.83, 95% CI [0.42, 1.24], *p* < 0.001, and Figure 3A; Supporting Information S1: Supporting Information 10 Table S8) in the CAI group. No clear asymmetries were identified (Supporting Information S1: Supporting Information 8 Figure S1).

No significant between‐group difference was observed in prelanding knee flexion angle (*p* > 0.05 and Figure 3A; Supporting Information S1: Supporting Information 10 Table S8). The asymmetry was only identified for knee flexion angle (Supporting Information S1: Supporting Information 8 Figure S1). Only for knee flexion angles, we excluded the work of Li et al. [21] and the between‐study heterogeneity decreased from 54% to 0% (Supporting Information S1: Supporting Information 9 Table S7). The pooled data showed no significant group differences in knee flexion angle and ROM after landing (*p* > 0.05 and Figure 3A; Supporting Information S1: Supporting Information 10 Table S8). The asymmetry was identified only for knee flexion angle (Supporting Information S1: Supporting Information 8 Figure S1). Only for knee flexion angle, we excluded the study of Li et al. [21] and the between‐study heterogeneity decreased from 68% to 0% (Supporting Information S1: Supporting Information 9 Table S7).

No significant group differences were found in hip flexion angles in both phases (*p* > 0.05 and Figure 3A; Supporting Information S1: Supporting Information 10 Table S8). No clear asymmetries were identified except for the prelanding hip flexion angle (Supporting Information S1: Supporting Information 8 Figure S1), and the exclusion of individual studies did not trigger a significant change in the overall results in sensitivity analyses.

#### 3.4.3 Kinetics

Meta‐analysis showed that CAI had no statistically significant effect on ankle peak eversion moment and plantarflexion moment (*p* > 0.05, Figure 3B; Supporting Information S1: Supporting Information 10 Table S8). No clear asymmetries were identified (Supporting Information S1: Supporting Information 8 Figure S1). The exclusion of individual studies did not trigger a significant change in sensitivity analyses.

Only the knee peak extension moment showed a significant change after removing the article written by Liu [53] (Supporting Information S1: Supporting Information 9 Table S7). The pooled data showed a medium effect (SMD = 0.71, 95% CI [0.32, 1.11], *p* < 0.001, and Figure 3B; Supporting Information S1: Supporting Information 10 Table S8) for increased peak knee extension moment. No clear asymmetries were identified (Supporting Information S1: Supporting Information 8 Figure S1).

### 3.5 Qualitative Synthesis

A number of other biomechanical variables were reported across all three lower limb joints, all three planes of motion, and during different phases of the landing task. However, most were reported in a single study and therefore could not be pooled (Supporting Information S1: Supporting Information 3 Table S2–Supporting Information 5 Table S4).

### 3.6 Subgroup Analysis

Pooled data showed a large effect for increased PL latency under the unexpected condition (ES = 1.68 and 95% CI [1.11, 2.24]) [23, 28, 34] and no difference under the expected condition (ES = 0.40 and 95% CI [−0.12, 0.91]) [24, 34] in patients with CAI compared with those in the control. Similarly, there was a medium effect for increased sCCI in prelanding (sCCI1) under the unexpected condition (ES = 0.53 and 95% CI [0.04, 1.01]) [23, 28, 34] and no difference under the expected condition (ES = 0.07 and 95% CI [−0.39, 0.54]) [25, 34] for patients with CAI. In addition, a medium effect for increased ankle inversion angle in postlanding (AI2) was found under the unexpected condition (ES = 0.50 and 95% CI [0.18, 0.82]) [19, 22, 34, 35], whereas no difference was found under the expected condition (ES = 0.20 and 95% CI [−0.17, 0.58]) [26, 34, 35, 53] (Supporting Information S1: Supporting Information 11 Table S9).

However, increased sCCI in postlanding (sCCI2), ankle inversion angle in prelanding (AI1), knee flexion angle in prelanding (KF1), knee flexion angle in postlanding (KF2), and knee‐flexion ROM in postlanding (KFROM2) had no difference under the unexpected condition (sCCI2: ES = 0.21 and 95% CI [−0.33, 0.75] [23, 34]; AI1: ES = 0.07 and 95% CI [−0.27, 0.40] [22, 23, 34, 35]; KF1: ES = 0.14 and 95% CI [−0.28, 0.56] [22, 35]; KF2: ES = 0.14 and 95% CI [−0.28, 0.56] [22, 35]; and KFROM2: ES = −0.04 and 95% CI [−0.63, 0.55] [35]) and a small‐to‐medium effect under the expected condition (sCCI2: ES = 0.57 and 95% CI [0.09, 1.04] [25, 34]; AI1: ES = 0.43 and 95% CI [0.06, 0.81] [21, 34, 35]; KF1: ES = 0.54 and 95% CI [0.10, 0.98] [21, 35]; KF2: ES = 0.77 and 95% CI [0.33, 1.22] [21, 35]; and KFROM2: ES = 0.41 and 95% CI [0.01, 0.81] [21, 35, 53]) for patients with CAI (Supporting Information S1: Supporting Information 11 Table S9). No between‐group differences were found for all the other parameters.

## 4 Discussion

The study aims to explore the current evidence for lower limb biomechanics in patients with CAI in comparison with those in healthy controls. Our study found that participants with CAI presented altered motor control patterns, displaying greater latency of PL muscle, sagittal muscle activation of ankle joint, ankle inversion angles, and knee extension moment during the LID task than control participants. Moreover, subgroup analyses revealed that the anticipation of disturbance could affect the motion strategy in CAI, notably in terms of ankle muscle activity, ankle frontal kinematics, and knee sagittal kinematics.

### 4.1 Motor Control in the Prelanding and Postlanding Phases

Before ground contact, we found that only the TA activity was higher in CAI patients than in controls; this finding coincided with previous results [11, 22]. The main role of TA is to control the dorsiflexion and inversion of ankle complex, keeping it in a relatively stable packed position [22]. Although a number of studies have already reported the functional deficits in PL, which may predispose individuals with CAI to recurrent lateral ankle injury [11, 23, 55, 56, 57, 58], the increased preactivation of TA might indicate that the reflexive activation of the primary evertor musculature may be unaffected but that an altered motor control strategy could manifest in individuals that develop CAI [56]. The possible mechanism of the varying activity of TA may be that patients with CAI develop a neuromuscular feedforward mechanism to provide dynamic joint stabilization [59, 60]. These patterns differ from regular proprioceptive feedback and could output anticipatory movement through the learning effects of the CNS [61].

We found that in the postlanding phase, PL activation was delayed and the sCCI and ankle inversion angle increased in the CAI group. Consistent with those of previous studies, the pooled results of our work revealed the delayed activation of PL in the CAI group [11, 34, 62, 63, 64]. The potential damage to the sensory receptors within lateral ankle ligaments in CAI would incapacitate the gamma motoneuron system and reduce sensitivity to quick peroneal muscle extension [14]. This damage may change spinal‐level motor control in patients with CAI and adversely affect feedback neuromuscular control in the lateral ankle muscles [34]. Consequently, disruptions in the gamma motoneuron loop would lead to reductions in alpha motor unit activation and prolong reaction times to aberrant ankle positioning [65, 66]. However, the previously published meta‐analysis by Chan et al. [55], which compared joint biomechanics in patients with CAI and controls, did not find similar results. This discrepancy might be attributed to the difference between the landing task types that we investigated [34]. Although the previous review included results from unilateral drop landing and stop‐jump tests, our review concentrated on the difference between the neuromuscular control of CAI and controls during the LID test, which may be more challenging and able to simulate the real injury and adaptation mechanisms of patients with CAI than other tests.

In addition, our results revealed no difference in muscle activation amplitude of the ankle joint after landing between patients with CAI and controls, whereas a significant alteration was found in sCCI, which is indicative of the increased coactivation of TA and MG in CAI [34]. To our knowledge, no meta‐analysis has reported muscle coactivation parameters, likely because of the lack or poor quality of previously available data. Researchers have mentioned that the CNS produces and coordinates movement by simultaneously activating muscles with opposing actions or reducing the dimensionality of the activation of numerous muscles into synergies when facing rapid or high‐demand tasks, and populations with dyskinesia, such as CAI, would exhibit high levels of coactivation, which may help them avoid the degeneration of effector‐level control [23, 34, 67, 68, 69, 70]. The increased preactivation of TA before landing, ankle sagittal muscle coactivation after landing, and increased demand of ankle frontal movement found in the present study may tend to decrease the sagittal displacement in CAI [56] and account for the lack of a significant difference in ankle sagittal kinematics between CAI and controls. Although the EMG results showed that CAI would change the muscle activation strategy to adjust the position of ankle joint, such efforts may still be insufficient to reduce the risk of reinjury after IC [22]. In line with previous studies [19, 20, 21, 22, 23, 34, 35], our work found an increased ankle inversion angle after landing (2.41°) in CAI. This finding corresponds to their PL deficits, as well as the inversion requirement in LID tasks, which could lead to excessive ankle inversion and even recurrent LAS.

Furthermore, consistent with the works of Li et al. [21] and Moisan et al. [52], our study found that the postlanding knee extension moment increased. One conjecture for the increase is that the increase in ankle inversion is accompanied with an increase in the distance between the center of the knee and ground, thus extending the lever arm of the tibia, which requires a large knee extension moment to resist ground reaction forces (GRFs) [71]. The other conjecture is that a high knee flexion angle leads to an increasing demand for knee extension moment to maintain joint stiffness and stability [72]. Given that we only found a nonsignificant increase in the postlanding knee flexion angle (1.39°) of patients with CAI, the increased knee extension moment may be a strategy of them to reduce the difference between their proximal segment movement from that of controls. The ankle joint is placed in an unstable position during LID. In consideration of the kinetic chain, feedback motor control may manifest as changes in the positioning of the proximal segments to lower the center of gravity and provide dynamic stability after the IC [73, 74]. The funnel plot of the knee flexion angle before and after landing showed remarkable asymmetry, which indicates large bias. Therefore, the overall results need to be treated with caution.

In contrast to Chan et al. [55] and Jeon et al. [58], we found reduced but not significant PL activation during ground contact in patients with CAI compared with that in controls. Although a previous study also reported that individuals with CAI showed no differences in PL activation during an unexpected single‐leg LID, the lack of significance may be due to the large heterogeneity and risk of publication bias among the current pooled studies. Therefore, the current results need to be interpreted with caution. Meanwhile, in contrast to previous studies, our work did not find significant alterations in hip joint biomechanics between patients with CAI and controls [22, 35, 55]. This situation is potentially due to the limited available data, such as hip biomechanical performance in unexpected trials [22] or GRFs in the pooled literature. The previous neglect of proximal joint functions might lead to the lack of hip performance data. Besides, the lack of GRFs is likely due to the difficulty in assessment of kinetics during the unexpected LID task and hence results in the lack of statistical power of hip joint biomechanics. Given consistent evidence that CAI alters interjoint coordination and elicits compensatory hip and knee mechanics during landing [30, 34, 68, 75, 76], future work could consider to examine the proximal‐joint biomechanics to clarify hip–knee–ankle coordination mechanics and motor control strategies of CAI.

### 4.2 Effect of Anticipation on Biomechanics in LID

An additional objective of this study is to examine anticipatory motor control strategies in ankle inversion perturbations during landing on a tilted surface. Subgroup analysis revealed that the anticipation of motion disturbance could affect the motion strategy of patients with CAI.

If the disturbance is expected, then individuals with CAI may increase their knee flexion angles to cushion the impact force in preparation for ground contact by using feedforward motor control [22, 34, 55], which may reflect the proximal dominant strategy of CAI [30]. The lack of changes in muscle activities showed that the descending pathway of PL may not have been completely damaged and the defects of PL could be recovered by central regulation to maintain stability and functional mobility [29]. The increased sCCI and unaltered ankle inversion angle after landing likely suggest that patients with CAI would reduce ankle inversion angle by increasing the coactivation of sagittal muscles. Furthermore, delayed activation, rather than the weakness of the quadriceps femoris, may lead to increased knee flexion angle after landing because the knee extension moment also increased [77]. However, given that the pooled evidence of the spatial–temporal characteristics of EMG and kinetics was insufficient, the underlying mechanism of the above strategy remains unclear.

We did not find differences in kinematics during LID under the unexpected condition. However, we did find an increase in sCCI before landing. This phenomenon might be explained by the limitation of the available data on the unexpected trials and biomechanics of proximal joints. Another mechanism for this phenomenon is likely the specific adaptation strategy developed by individuals with CAI, who are believed to develop a rigid and preprepared motion strategy from prior movement experience [78]. Such strategy can increase the stiffness of the lower limb joints and reserve the redundancy of mobility and synergistic muscle activation to prepare for the unexpected risk of injury [22, 68, 79]. In the post‐landing phase, the delayed activation of the PL and increased inversion of the ankle implied that the preincrease in sCCI may be insufficient to stabilize the ankle joint after accidental inverted perturbations, and the unexpected extension of the PL can induce its dysfunction easily. In addition, recent studies have found that patients with CAI may be unable to reweight other sensory inputs to compensate for decreased visual information [80]. Given that the unexpected, closed‐eye, and visual disruption tasks are similar, our result may be consistent with the findings for these tasks showing that the impaired somatosensory systems of patients with CAI could inhibit efficient and safe movement patterns when visual information is limited [76, 81]. For example, Ghislieri et al. [69] found that muscle synergy in CAI was lower than that in healthy controls when they performed the single‐leg‐stance task with closed eyes, and Lee et al. [81] found that individuals with CAI may exhibit increased ankle inversion in the landing–cutting task with visual disruption. Finally, although the pooled EMG evidence in our study is inadequate, previous studies have suggested that the proximal muscle of CAI would be activated in a compensatory manner during landing, and this phenomenon may contribute to the whole dynamic stability of the lower extremities [30, 67].

### 4.3 Clinical Implications and Future Research

The findings of this meta‐analysis have important clinical implications for the assessment and rehabilitation of individuals with CAI. First, clinicians could consider using comprehensive EMG indicators, such as muscle synergy, and increase investigations on proximal joints to assess neuromuscular control strategies in patients with CAI [30]. Muscle synergy analysis can provide an accurate understanding of the coordinated activation patterns of multiple muscles, which may help identify the underlying neuromuscular deficits in CAI [67, 68, 82]. Second, the anticipation of perturbations can substantially influence the neuromuscular control strategies of patients with CAI during LID tasks. Therefore, future rehabilitation training programs should incorporate elements of anticipation and inversion perturbances with landing to simulate real‐world conditions and improve the functional stability of patients with CAI to cope with potentially injurious positions [22, 31, 83].

### 4.4 Limitations

Our study is the first to provide a full map of the pooled analysis of lower extremity muscle activity, kinematics, and kinetics in CAI during a LID task, which is a realistic injury simulation and functional movement test. Moreover, we found an effect of the anticipation of inversion disturbance on the biomechanical performance of participants with CAI. However, several limitations should be acknowledged. First, the included studies exhibited significant methodological heterogeneity, particularly in terms of participant characteristics, testing protocols, and statistical methods. Although we attempted to control for these variables and heterogeneity decreased significantly after we conducted sensitivity analyses, they may still affect the robustness of the EMG findings. Second, due to limited data availability, we were unable to conduct subgroup analyses based on factors, such as jump height, inversion disturbance angle, and participant gender, although these factors mainly concentrate on a specific value. Third, current data on EMG and the kinetics of the proximal joint in patients with CAI during landing tasks are limited. Therefore, no pooled evidence can be formed to assist to analyze the kinematic results. Considering that researchers have reported that people with CAI exhibited neuromuscular variations in the proximal muscles [38, 67, 84] and altered hip–knee–ankle coordination [85] during landing tasks, it would be valuable to prioritize proximal‐joint biomechanics of CAI to comprehensively clarify the adaptational mechanisms of motor control system and optimize multijoint rehabilitation.

## 5 Conclusion

The findings highlight that relative to those in healthy controls, TA preactivation and ankle muscle coactivation are increased and joint angles and moments are altered in CAI. These changes may contribute to their increased risk of recurrent LAS. However, further standardized studies are required to validate these variations. We can thus further enhance our understanding of the neuromuscular control mechanisms in CAI and develop more effective rehabilitation strategies to improve the functional stability and quality of life of patients with CAI.

## Author Contributions

**Yixue Gong:** conceptualization, data curation, formal analysis, methodology, writing – original draft, writing – review and editing. **Menghan Xu:** investigation, formal analysis, writing – original draft. **Peng Chen:** methodology, writing – review and editing. **Xiaomei Hu:** methodology, writing – review and editing. **Wenxing Zhou:** methodology, formal analysis. **Lin Wang:** conceptualization, funding acquisition, project administration.

## Ethics Statement

The authors have nothing to report.

## Consent

The authors have nothing to report.

## Conflicts of Interest

The authors declare no conflicts of interest.

## Supporting information


Supporting Information S1

## Data Availability

All data analyzed during this study are included within the article and supplementary files.
