# Supplementary material for: Characteristics of Lower Extremity Kinematics, Kinetics, and Muscle Activity in Individuals With Chronic Ankle Instability During Landing With Expected and Unexpected Inversion Perturbations: A Systematic Review and Meta‐Analysis
Source: J Foot Ankle Res. 2025 Sep 8;18(3):e70082. doi: 10.1002/jfa2.70082 (PMC12417340; doi:10.1002/jfa2.70082)
Supplement: Supplementary file 1 — Supporting Information S1 [file JFA2-18-e70082-s001.docx]

**Supporting Information**

**Article Title****:** Characteristics of lower extremity kinematics, kinetics, and muscle activity in individuals with chronic ankle instability during landing with expected and unexpected inversion perturbations: A systematic review and meta-analysis.

**Table of Contents**

[Supporting Information 1 Table S1. Search strategies 3](#_Toc17154)

[Supporting Information 2. Newcastle-Ottawa quality assessment scale 7](#_Toc22507)

[Supporting Information 3 Table S2. Study outcomes for muscle activity 14](#_Toc25730)

[Supporting Information 4 Table S3. Study outcomes for joint kinematics 22](#_Toc18103)

[Supporting Information 5 Table S4. Study outcomes for joint kinetics 29](#_Toc2648)

[Supporting Information 6 Table S5. Inclusion criteria of studies included in the systematic review based on the recommendation of IAC 34](#_Toc14887)

[Supporting Information 7 Table S6. Summary of pooled evidence—electromyography 36](#_Toc20055)

[Supporting Information 8 Figure S1. The funnel plots of muscle activity, kinematics and kinetics 38](#_Toc11234)

[Supporting Information 9 Table S7. Results of sensitivity analyses 41](#_Toc30241)

[Supporting Information 10 Table S8. Summary of pooled evidence—kinematics and kinetics 42](#_Toc6963)

[Supporting Information 11 Table S9. The results from the changes in heterogeneity in each subgroup analysis 45](#_Toc23360)

**Supporting Information 1 Table S1.** Search strategies

Databases: PubMed, Web of Science, EMBASE, Cochrane Library, Scopus.

Search data: 30 November 2024.

Key words:

1. Ankle-associated words: (ankle [MeSH] OR ankle joint [MeSH] OR lateral ligament* OR talo* OR tibiofib* OR tibio-fib* OR lower extremity [MeSH] OR lower limb ) [Titile/Abstract]
2. Injury-associated words: (joint instability [MeSH] OR joint instability [Text Word] OR ankle injuries [MeSH] OR instabilit* OR unstable OR sprain* OR strain* OR tear* OR laxity) [Titile/Abstract]
3. Landing-associated words: (landing OR drop OR drop-landing OR jump* OR countermovement jump) [Titile/Abstract]
4. Perturbance-associated words: (unexpect* OR expect* OR anticipat* OR disturbance OR perturba* OR slope OR elevat* OR inclin* OR tilt* OR inver* OR supin*)
5. Biomechanics-associated words: (electromyography OR emg OR muscle OR kinematic* OR kinetic* OR biomechanic* OR neuromuscular control OR motor control OR joint-coupling)

Search strategy: 1 AND 2 AND 3 AND 4 AND 5

| **PubMed** | | |
| --- | --- | --- |
| **Concept** | **Query** | **Hits** |
| #1 | (ankle [MeSH]) OR (ankle joint [MeSH]) OR (lateral ligament*[Title/Abstract]) OR (talo*[Title/Abstract]) OR (tibiofib*[Title/Abstract]) OR (tibio-fib*[Title/Abstract]) OR (lower extremity [MeSH]) OR (lower limb[Title/Abstract]) | 246,088 |
| #2 | (joint instability [MeSH]) OR (joint instability [Text Word]) OR (ankle injur*[MeSH]) OR (instabilit*[Title/Abstract]) OR (unstable[Title/Abstract]) OR (sprain*[Title/Abstract]) OR (strain*[Title/Abstract]) OR (tear*[Title/Abstract]) OR (laxity[Title/Abstract]) | 1,248,598 |
| #3 | (landing[Title/Abstract]) OR (drop[Title/Abstract]) OR (drop-landing[Title/Abstract]) OR (jump*[Title/Abstract]) OR (countermovement jump[Title/Abstract]) | 143,888 |
| #4 | (unexpect*) OR (expect*) OR (anticipat*) OR (disturbance) OR (perturba*) OR (slope) OR (elevat*) OR (inclin*) OR (tilt*) OR (inver*) OR (supin*) | 2,908,125 |
| #5 | (electromyography) OR (emg) OR (muscle) OR (kinematic*) OR (kinetic*) OR (biomechanic*) OR (neuromuscular control) OR (motor control)) OR (joint-coupling) | 2,405,850 |
| #6 | #1 AND #2 AND #3 AND #4 AND #5 | **173** |

| **Wed of Science** | | |
| --- | --- | --- |
| **Concept** | **Query** | **Hits** |
| #1 | ((((((((TS=(electromyography)) OR TS=(emg)) OR TS=(muscle)) OR TS=(kinematic*)) OR TS=(kinetic*)) OR TS=(biomechanic*)) OR TS=("neuromuscular control")) OR TS=("motor control")) OR TS=(joint-coupling) and Preprint Citation Index (Exclude – Database) | 2223210 |
| #2 | ((((((((((TS=(unexpect*)) OR TS=(expect*)) OR TS=(anticipat*)) OR TS=(disturbance)) OR TS=(perturba*)) OR TS=(slope)) OR TS=(elevat*)) OR TS=(inclin*)) OR TS=(tilt*)) OR TS=(inver*)) OR TS=(supin*) and Preprint Citation Index (Exclude – Database) | 4906037 |
| #3 | ((((TS=(landing)) OR TS=(drop)) OR TS=(drop-landing)) OR TS=(jump*)) OR TS=("countermovement jump") and Preprint Citation Index (Exclude – Database) | 1530177 |
| #4 | (((((((TS=(joint instability)) OR TS=(ankle injur*)) OR TS=(instabilit*)) OR TS=(unstable)) OR TS=(sprain*)) OR TS=(strain*)) OR TS=(tear*)) OR TS=(laxity) and Preprint Citation Index (Exclude – Database) | 2492543 |
| #5 | ((((((((TS=(ankle)) OR TS=(ankle joint)) OR TS=("lateral ligament*")) OR TS=(talo*)) OR TS=(tibiofib*)) OR TS=(tibio-fib*)) OR TS=("lower extremity")) OR TS=("lower limb")) NOT (SILOID==("PPRN")) | 145820 |
| #6 | #1 AND #2 AND #3 AND #4 AND #5 and Preprint Citation Index (Exclude – Database) | **518** |

| **EMBASE** | | |
| --- | --- | --- |
| **Concept** | **Query** | **Hits** |
| #1 | TI ( ankle or "ankle joint" or "lateral ligament*" or talo* or tibiofib* or tibio-fib* or "lower extremity" or "lower limb" ) AND AB ( ankle or "ankle joint" or "lateral ligament*" or talo* or tibiofib* or tibio-fib* or "lower extremity" or "lower limb" ) | 231,186 |
| #2 | TI ( "joint instability" or "ankle injur*" or instabilit* or unstable or sprain* or strain* or tear* or laxity ) AND AB ( "joint instability" or "ankle injur*" or instabilit* or unstable or sprain* or strain* or tear* or laxity ) | 1,419,042 |
| #3 | TI ( landing or drop or drop-landing or jump* or "countermovement jump" ) AND AB ( landing or drop or drop-landing or jump* or "countermovement jump" ) | 187,444 |
| #4 | TI ( unexpect* or expect* or anticipat* or disturbance or perturba* or slope or elevat* or inclin* or tilt* or inver* or supin* ) AND AB ( unexpect* or expect* or anticipat* or disturbance or perturba* or slope or elevat* or inclin* or tilt* or inver* or supin* ) | 3,478,933 |
| #5 | TI ( electromyography or emg or muscle or kinematic* or kinetic* or biomechanic* or "neuromuscular control" or "motor control" or joint-coupling ) AND AB ( electromyography or emg or muscle or kinematic* or kinetic* or biomechanic* or "neuromuscular control" or "motor control" or joint-coupling ) | 1,693,868 |
| #6 | #1 AND #2 AND #3 AND #4 AND #5 | **221** |

| **Cochrane Library** | | |
| --- | --- | --- |
| **Concept** | **Query** | **Hits** |
| #1 | ("ankle" OR "ankle joint"):ti,ab,kw OR ("lateral ligament"):ti,ab,kw OR (talo* OR tibiofib* OR tibio-fib*):ti,ab,kw OR ((lower extremit*) OR (lower limb*)):ti,ab,kw | 39525 |
| #2 | ("joint instability" OR instabilit* OR unstable):ti,ab,kw OR ((ankle injur*) OR sprain* OR strain* OR tear* OR laxity):ti,ab,kw | 46844 |
| #3 | (landing OR drop* OR jump* OR "countermovement jump"):ti,ab,kw | 51827 |
| #4 | (unexpect* OR expect* OR anticipat*) OR (disturbance OR perturba*) OR (slope OR elevat* OR inclin* OR tilt* OR inver* OR supin*) | 209232 |
| #5 | (electromyograph* OR emg OR muscle) OR (kinematic* OR kinetic*) OR (biomechanic* OR "neuromuscular control" OR "motor control") OR (joint-coupling) | 133449 |
| #6 | #1 AND #2 AND #3 AND #4 AND #5 | **119** |

| **Scopus** | | |
| --- | --- | --- |
| **Concept** | **Query** | **Hits** |
| #1 | TITLE-ABS-KEY ( ankle OR "ankle joint" OR "lateral ligament*" OR talo* OR tibiofib* OR tibio-fib* OR "lower extremity" OR "lower limb" ) | 313,725 |
| #2 | TITLE-ABS-KEY ( "joint instability" OR "ankle injur*" OR instabilit* OR unstable OR sprain* OR strain* OR tear* OR laxity ) | 3,481,531 |
| #3 | TITLE-ABS-KEY ( landing OR drop OR drop-landing OR jump* OR "countermovement jump" ) | 826,135 |
| #4 | TITLE-ABS-KEY ( unexpect* OR expect* OR anticipat* OR disturbance OR perturba* OR slope OR elevat* OR inclin* OR tilt* OR inver* OR supin* ) | 7,656,733 |
| #5 | TITLE-ABS-KEY ( electromyography OR emg OR muscle OR kinematic* OR kinetic* OR biomechanic* OR "neuromuscular control" OR "motor control" OR joint-coupling ) | 4,467,962 |
| #6 | #1 AND #2 AND #3 AND #4 AND #5 | **306** |

**Supporting Information 2.** Newcastle-Ottawa quality assessment scale

**NEWCASTLE - OTTAWA QUALITY ASSESSMENT SCALE**

**CASE CONTROL STUDIES**

Note: A study can be awarded a maximum of one star for each numbered item within the Selection and Exposure categories. A maximum of two stars can be given for Comparability.

**Selection**

1) Is the case definition adequate?

a) yes, with independent validation **🟑**

b) yes, eg record linkage or based on self reports

c) no description

2) Representativeness of the cases

a) consecutive or obviously representative series of cases **🟑**

b) potential for selection biases or not stated

3) Selection of Controls

a) community controls **🟑**

b) hospital controls

c) no description

4) Definition of Controls

a) no history of disease (endpoint) **🟑**

b) no description of source

**Comparability**

1) Comparability of cases and controls on the basis of the design or analysis

a) study controls for _______________ (Select the most important factor.) **🟑**

b) study controls for any additional factor **🟑** (This criteria could be modified to indicate specific control for a second important factor.)

**Exposure**

1) Ascertainment of exposure

a) secure record (eg surgical records) **🟑**

b) structured interview where blind to case/control status **🟑**

c) interview not blinded to case/control status

d) written self report or medical record only

e) no description

2) Same method of ascertainment for cases and controls

a) yes **🟑**

b) no

3) Non-Response rate

a) same rate for both groups **🟑**

b) non respondents described

c) rate different and no designation

**NEWCASTLE - OTTAWA QUALITY ASSESSMENT SCALE**

**COHORT STUDIES**

Note: A study can be awarded a maximum of one star for each numbered item within the Selection and Outcome categories. A maximum of two stars can be given for Comparability

**Selection**

1) Representativeness of the exposed cohort

a) truly representative of the average _______________ (describe) in the community **🟑**

b) somewhat representative of the average ______________ in the community **🟑**

c) selected group of users eg nurses, volunteers

d) no description of the derivation of the cohort

2) Selection of the non exposed cohort

a) drawn from the same community as the exposed cohort **🟑**

b) drawn from a different source

c) no description of the derivation of the non exposed cohort

3) Ascertainment of exposure

a) secure record (eg surgical records) **🟑**

b) structured interview **🟑**

c) written self report

d) no description

4) Demonstration that outcome of interest was not present at start of study

a) yes **🟑**

b) no

**Comparability**

1) Comparability of cohorts on the basis of the design or analysis

a) study controls for _____________ (select the most important factor) **🟑**

b) study controls for any additional factor **🟑** (This criteria could be modified to indicate specific control for a second important factor.)

**Outcome**

1) Assessment of outcome

a) independent blind assessment **🟑**

b) record linkage **🟑**

c) self report

d) no description

2) Was follow-up long enough for outcomes to occur

a) yes (select an adequate follow up period for outcome of interest) **🟑**

b) no

3) Adequacy of follow up of cohorts

a) complete follow up - all subjects accounted for **🟑**

b) subjects lost to follow up unlikely to introduce bias - small number lost - > ____ % (select an adequate %) follow up, or description provided of those lost) **🟑**

c) follow up rate < ____% (select an adequate %) and no description of those lost

d) no statement

**CODING MANUAL FOR CASE-CONTROL STUDIES**

***SELECTION***

1. **Is the Case Definition Adequate?**
2. Requires some independent validation (e.g. >1 person/record/time/process to extract information, or reference to primary record source such as x-rays or medical/hospital records)
3. Record linkage (e.g. ICD codes in database) or self-report with no reference to primary record
4. No description
5. **Representativeness of the Cases**
6. All eligible cases with outcome of interest over a defined period of time, all cases in a defined catchment area, all cases in a defined hospital or clinic, group of hospitals, health maintenance organisation, or an appropriate sample of those cases (e.g. random sample)
7. Not satisfying requirements in part (a), or not stated.
8. **Selection of Controls**

This item assesses whether the control series used in the study is derived from the same population as the cases and essentially would have been cases had the outcome been present.

1. Community controls (i.e. same community as cases and would be cases if had outcome)
2. Hospital controls, within same community as cases (i.e. not another city) but derived from a hospitalised population
3. No description
4. **Definition of Controls**
5. If cases are first occurrence of outcome, then it must explicitly state that controls have no history of this outcome. If cases have new (not necessarily first) occurrence of outcome, then controls with previous occurrences of outcome of interest should not be excluded.
6. No mention of history of outcome

***COMPARABILITY***

1. **Comparability of Cases and Controls on the Basis of the Design or Analysis**

A maximum of 2 stars can be allotted in this category

Either cases and controls must be matched in the design and/or confounders must be adjusted for in the analysis. Statements of no differences between groups or that differences were not statistically significant are not sufficient for establishing comparability. Note: If the odds ratio for the exposure of interest is adjusted for the confounders listed, then the groups will be considered to be comparable on each variable used in the adjustment.

There may be multiple ratings for this item for different categories of exposure (e.g. ever vs. never, current vs. previous or never)

Age = , Other controlled factors =

***EXPOSURE***

1. **Ascertainment of Exposure**

Allocation of stars as per rating sheet

1. **Non-Response Rate**

Allocation of stars as per rating sheet

**CODING MANUAL FOR COHORT STUDIES**

***SELECTION***

1. **Representativeness of the Exposed Cohort**

Item is assessing the representativeness of exposed individuals in the community, not the representativeness of the sample of women from some general population. For example, subjects derived from groups likely to contain middle class, better educated, health oriented women are likely to be representative of postmenopausal estrogen users while they are not representative of all women (e.g. members of a health maintenance organisation (HMO) will be a representative sample of estrogen users. While the HMO may have an under-representation of ethnic groups, the poor, and poorly educated, these excluded groups are not the predominant users users of estrogen).

Allocation of stars as per rating sheet

1. **Selection of the Non-Exposed Cohort**

Allocation of stars as per rating sheet

1. **Ascertainment of Exposure**

Allocation of stars as per rating sheet

1. **Demonstration That Outcome of Interest Was Not Present at Start of Study**

In the case of mortality studies, outcome of interest is still the presence of a disease/ incident, rather than death. That is to say that a statement of no history of disease or incident earns a star.

***COMPARABILITY***

1. **Comparability of Cohorts on the Basis of the Design or Analysis**

A maximum of 2 stars can be allotted in this category

Either exposed and non-exposed individuals must be matched in the design and/or confounders must be adjusted for in the analysis. Statements of no differences between groups or that differences were not statistically significant are not sufficient for establishing comparability. Note: If the relative risk for the exposure of interest is adjusted for the confounders listed, then the groups will be considered to be comparable on each variable used in the adjustment.

There may be multiple ratings for this item for different categories of exposure (e.g. ever vs. never, current vs. previous or never)

Age = , Other controlled factors =

***OUTCOME***

1. **Assessment of Outcome**

For some outcomes (e.g. fractured hip), reference to the medical record is sufficient to satisfy the requirement for confirmation of the fracture. This would not be adequate for vertebral fracture outcomes where reference to x-rays would be required.

1. Independent or blind assessment stated in the paper, or confirmation of the outcome by reference to secure records (x-rays, medical records, etc.)
2. Record linkage (e.g. identified through ICD codes on database records)
3. Self-report (i.e. no reference to original medical records or x-rays to confirm the outcome)
4. No description.
5. **Was Follow-Up Long Enough for Outcomes to Occur**

An acceptable length of time should be decided before quality assessment begins (e.g. 5 yrs. for exposure to breast implants)

1. **Adequacy of Follow Up of Cohorts**

This item assesses the follow-up of the exposed and non-exposed cohorts to ensure that losses are not related to either the exposure or the outcome.

Allocation of stars as per rating sheet

| **Supporting Information 3 Table S2.** Study outcomes for muscle activity | | | |
| --- | --- | --- | --- |
| Study | Landing task | Muscle Activity, Pre-landing | Muscle Activity, Post-landing |
| Gutierrez 2012 | Drop-jump testing | The EMG data were normalized to the maximum activity during the unknown, non-supinating trials in each respective muscle.  The AI group had significantly more activity of the PL in pre-landing (before IC 200ms) than control group (P = .009).  No statistically significant differences in TA. | Post-landing (after IC 200ms) has significant increases in PL reactive EMG area (F1,42 = 12.133; P = .001）.  No statistically significant differences in TA. |
| Han 2023 | single-leg drop-landing | NR | NR |
| Knight 2011 | Dingle-leg drop-landing | Latency was defined as the time from contact of the fulcrum with the landing area (initiation of inversion moment) to the time of muscle activity exceeding 5 SD from a baseline muscle activity.  Mean latency of PL is 45.08 ± 11.71 ms, no significant difference among LAS and control groups (p = .990).  Mean latency of PB is 54.40 ± 16.70 ms, no significant difference between groups (p = .781). | NR |
| Lee 2023 | Single-leg drop-landing | The EMG amplitudes from 3 seconds of quiet standing position were defined as the reference values of EMG data as quiet standing. Then, the EMG data were zeroed to baseline, rectified, and band-pass filtered (10-500 Hz).  CAI subjects showed 11% more TA activation 120-ms before to 140-ms after IC compared to controls, and 3% more GM activation from 200-ms before the IC to the IC. | CAI subjects showed 8.5% more PL activation from 15-ms to 200-ms after IC, 5% more MG activation from 80-ms before to 15-ms after IC, 5% more GX activation from 140-ms to 200-ms after IC, 8.5% more MG activation from IC to 60-ms after IC, and 25% more VL activation from 50-ms before to 200-ms after IC. |
| Levin 2015 | The left leg (control group) or the affected leg (CAI group) in non-inverting condition (0%LOCK, EXP), 50% non-inverting condition (50%LOCK, UE), 50% inverting condition (50%INV, UE) or 100% inverting condition (100%INV, EXP) of the landing trials | The RMS of the rectified averaged EMG time series were calculated at three prefixed time windows within a 500 ms period of interest, ranging from-200 ms prior to IC (pre-landing) to 300 ms after IC (post-landing). The first reflex window (+30 to +60 ms) corresponded to SLR and the second window (+80 to +135 ms) corresponded to LLR.  The resulting RMS values of each muscle were normalized with respect to the RMS of the EMG signal within the 500 ms window of interest during the LOCK, defined as baseline RMS.  As compared to controls, CAI generally showed higher levels of activity in the pre-landing. Significant differences between groups in the levels of preparatory muscle activity were observed for the PL and MG on the non-inverting side during conditions with either 100% chance of inversion [PL controls: M = 1.14 ± 0.23 vs. CAIs = 1.50± 0.38; MG controls: M = 1.65 ± 0.41 vs. CAIs: 2.09 ± 0.22; both p = 0.034] or 50% chance of inversion [PL controls: M = 1.15 ± 0.38 vs. CAIs = 1.76± 0.61, p = 0.027].  For subjects with CAI, significant main effects of conditions were found but only for the MG on the inverting side [ x 2 (9,2) = 6.22, p = 0.044]. | CAI group showed lower activation of PL (p＜0.022), MG (p＜0.022) and GX (p = 0.022) in 100% LOCK condition.  Similar differences existed in 50% LOCK (PL: 1.35 ± 0.46, MG: 0.66 ± 0.22, p ≤ 0.008) and 50% INV (PL: 1.49 ± 0.89, MG: 0.93 ± 0.38, p ≤ 0.022) conditions in SLR phase.  Significant group differences in LLR phase for TA in 100% INV condition (controls: 1.33 ± 0.71, CAIs: 0.85 ± 0.26, p = 0.010).  CON showed f anticipatory-induced larger SLR of MG (p=0.021) and CAI group showed lower SLR of PL (p=0.008) as well as larger LLR of GX (p=0.008) in 100%LOCK condition.  No significant group differences in EMG RMS normalized values for SLR and LLR were found for the muscles on the non-inverting side (p ≥0.05). |
| Li 2018a | A double-leg landing with test limb landing: non-test limbs on the flat platforms and CAI limb on inverted platforms | The direct current offset was removed by subtracting the mean value of the EMG signal of the entire trial from the raw data. To normalize and compare the EMG data across participants, MVIC tests were conducted. A 5-s EMG signal (one trial) was captured for each muscle MVIC test and a 30-s break was taken between the tests.  CAI group exhibited significantly lower PL activation, greater LG, and a tendency of increased VL activation (p=0.083). Significantly greater ankle CCI in the frontal plane was displayed by CAI individuals (P=0.013).  No statistically significant differences were noted in TA, RF and BF activation, as well as CCR Q/H (quadriceps to hamstring) and sagittal CCI. | CAI individuals exhibited greater activation for TA, RF, and VL, and a tendency of reduced BF activation (p=0.068).  Greater CCI of ankle muscles in both sagittal and frontal planes for CAI compared to controls. CCR Q/H was significantly greater for the CAI group.  No temporal variables were significantly different (p=0.106–0.617).  No statistically significant differences were noted in PL and LG activation. |
| Li 2018b | A double-leg landing with test limb landing: non-test limbs on the flat platforms and CAI limb on inverted platforms | NR | NR |
| Liu 2013 | A single-leg drop-landing on a flat surface, an inverted surface and a combined surface of inversion and PF | NR | NR |
| Moisan 2020 | unilateral side jump landing (SIDE) and unilateral drop landing on three surfaces [even (DROP), unstable (FOAM) and laterally 25° inclined (WEDGE)] | EMG data were quantified during the post-landing and the pre-landing (from heel off to IC) phases and were normalized to 0 to 100% of each phase.  For the SIDE task, CAI group exhibited decreased BF muscle activity from 73 to 100% (p < 0.01) of the pre-landing phases. GM muscle activity decreased from 0 to 5% (p = 0.01) of the pre-landing phase.  For the DROP task, decreased VL muscle activity from 80 to 88% (p = 0.01) of the pre-landing phase.  For the WEDGE task, increased BF muscle activity from 39 to 40% (p = 0.02) of the pre-landing phase. Decreased muscle activity of the GM from 87 to 100% (p < 0.01) and PL from 92 to 94% (p = 0.02) and 96 to 97% (p = 0.02) of the pre-landing. | For the SIDE task, CAI group exhibited decreased BF muscle activity from 0 to 12% (p = 0.01) of the post-landing phases.  For the WEDGE task, increased BF muscle activity from 33 to 56% (p = 0.02) of the post-landing phase. |
| Simpson 2019 | single-leg drop-landing | Average EMG amplitudes during 200ms in pre-landing and 200ms in post-landing were normalized to each participant highest 100ms average of a 3-s MVIC for each muscle. Latency of the PL and PB was determined from the rectified EMG signal as the time in ms from when the vertical component of the ground reaction force exceeded 15N, which coincided with the initiation of the inversion perturbation, to the point where muscle activity exceeded 5 SD above the averaged 200 ms pre-landing muscle activity.  Results revealed no significant condition by group interactions for latency of the PL and PB, or for any of the pre- and post-initial contact muscle activity variables (p > 0.05).  A significant group main effect for PL latency was observed, with the CAI group exhibiting significantly longer PL latency compared to the control group (p < 0.001; d =1.20).  Significant condition main effects were also observed for PL and PB latency, with significantly reduced PL (p=0.004; d =0.90) and PB (p= 0.011; d = 0.66) latency during EXP when compared to UE.  No statistically significant differences were noted in activation of TA and MG, as well as sagittal plane CCI and frontal plane CCI. | Significant condition main effects were found for TA and frontal plane CCI. Significantly greater TA activity (p=0.009; d =0.40) and frontal plane CCI (p < 0.001; d = 0.97) were found in post-landing during the EXP trial in comparison to the UE trial.  There were no other significant interactions or main effects for pre- or post-landing EMG variables.  No statistically significant differences were noted in activation of MG, PL and PB, as well as sagittal plane CCI. |
| Song 2019 | single-leg drop-landing | EMG data were collected at 1,000 Hz. After a fourth-order zero-phase low-pass filter (10 Hz) and baseline subtraction, RMS was calculated. For maximum isometric contraction, RMS of the 1-second activation value during a 5-second contraction was calculated, and EMG activation percentage was obtained by dividing EMG activation by MVIC activation.  In pre-landing, GAS activation on the injured side of the FAI group was significantly higher than on the healthy side (P < 0.05). TA, PL, and SOL activation showed no significant differences between the injured and healthy sides (P > 0.05). PL activation on the injured side of the FAI group was significantly higher than in the healthy control group (P < 0.05), but TA, GAS, and SOL activation showed no significant differences between the injured side of the FAI group and the healthy control group (P > 0.05). | In post-landing, PL activation on the injured side of the FAI group was significantly lower than that on the healthy side (P < 0.05), while there were no significant differences in TA, GAS, and SOL activation between the injured and healthy sides (P > 0.05). No significant differences were found between the injured and healthy sides for all four muscles (P > 0.05). |
| Watabe 2021a | single-leg landing then cutting | The latency of the PL was determined from rectified EMG signals as time in milliseconds from when the vertical component of the ground reaction force exceeded 10 N, which coincided with the initiation of the inversion perturbation, to the point where muscle activity exceeded 5 SD above the average 200 ms pre-landing muscle activity.  The PL latency was significantly shorter in the CON groups compared with that in the CAI group when the landing board was tilted to the right (p = 0.004) and left (p = 0.001). No significant difference was observed in the PL latency of the CON groups in all tasks. No other significant interactions or main effects were observed for the pre- landing EMG variables, sagittal and frontal plane CI (p > 0.05). | NR |
| Watabe 2021b | single-leg landing | The latency of the PL was determined from rectified EMG signals as time in ms from when the vertical component of the ground reaction force exceeded 10 N, which coincided with the initiation of the inversion perturbation, to the point where the muscle activity exceeded 5 SD above the average 200 ms pre-landing muscle activity. The response time of the PL muscle was determined three times for each task, and the average of the three reactions was calculated.  No significant difference was observed in the muscle activity at 200-ms pre-landing between the CAI, and control groups (p > 0.05). | The PL latency was significantly delayed in the CAI group compared to the control group during inversion landing (p = 0.002) and normal landing (p =0.005).  The muscle activity of PL at 200-ms post-landing decreased significantly in the CAI group compared to the controls during inversion landing (p = 0.049).  The muscle activity of the 200-ms post-landing frontal plane CCI was significantly increased in the CAI group compared to that in the control group during inversion landing (p =0.018). |
| **Abbreviations:** CAI = chronic ankle instability group; CON = control group; AI = ankle instability; LAS = lateral ankle sprains group; FAI = functional ankle instability group; EMG = electromyogram; IC = initial contact; SD = standard deviations; NR = not reported; PL = peroneus longus; PB = peroneus brevis; TA = tibialis anterior; GX = gluteus maximus; GM = gluteus medius; MG = gastrocnemius medialis; LG = gastrocnemius lateralis; VL = vastus lateralis; RF = rectus femoris; BF = biceps femoris; UE = unexpected; EXP = expected; RMS = root mean square; SLR = short latency response; LLR = long latency response; M = mean±SD; MVIC = maximum voluntary isometric contraction; CCR = co-contraction ratio; CCI = co-contraction index; PF = plantarflexion; N = newton. | | | |

| **Supporting Information 4 Table S3.** Study outcomes for joint kinematics | | | | |
| --- | --- | --- | --- | --- |
| Study | Outcome Variables | Ankle Kinematics | Knee Kinematics | Hip Kinematics |
| Gutierrez 2012 | Pre-landing (before IC 200ms)  Post-landing (after IC 200ms)  IC = the instant of touchdown = vGRF＞0 N (2 SD above baseline noise) | *Pre-landing:*  No statistically significant differences were reported in ankle PF, inversion and ADD.  *Post-landing:*  On average, after IC 200ms, the platform caused approximately 5° of ankle PF, 8°of ADD, and 4.5° of inversion.  All groups caused increased maximum ankle PF (F1,42 = 71.287; P < .001), ADD (F1,42 = 25.536; P < .001), and inversion (F1,42 = 0.290; P < .001) after IC 50ms. | NR | NR |
| Han 2023 | Pre-landing (IC)  Post-landing (after IC 200ms)  IC = vGRF > 15 N | *Pre-landing:*  Total ankle displacement and maximum inversion velocity were increased at IC under the UE condition (p<0.01), with a decreased inversion angle at IC (p<0.01). CAI patients consistently displayed a greater maximum inversion angle compared to controls (p=0.02).  *Post-landing:*  Significant group by condition interactions for total ankle displacement in the frontal plane (p<0.01) and maximum inversion velocity (p=0.01).  CAI patients and controls showed increased total ankle displacement in the frontal plane under the UE condition (p<0.01).  CAI patients displayed increased maximum inversion velocity under the UE condition (p<0.01), and had a greater maximum inversion angle (p=0.02) and greater total ankle displacement in the frontal plane and maximum inversion velocity than controls. | No significant group (knee flexion angle at IC, maximum knee flexion angle and knee flexion displacement) by condition interactions. | *Pre-landing:*  No statistically significant difference was noted in hip flexion Angle at IC.  *Post-landing:*  Main effects for the condition were present for maximum hip flexion angle and displacement (p<0.01). |
| Knight 2011 | Latency**:** the time from contact of the fulcrum with the landing area (initiation of inversion moment) to the time of muscle activity exceeding 5 SD from a baseline muscle activity, which was taken 200 ms before landing.  The time to maximum inversion (ms): the landing area to contact of the lateral border of the outer sole with the landing area. | The mean time to maximum inversion across CAI, LAS and control groups was 44.74 ± 17.12 ms. There was not a significant difference in the time to maximum inversion among the three injury groups (p = .123). | NR | NR |
| Lee 2023 | Pre-landing (before IC 200ms)  Post-landing (after IC 200ms)  IC = vGRF > 10N | ***Pre-landing:***  CAI subjects showed up to 4° more DF from 200-ms before to 80-ms after the IC. No significance was found in ankle inversion angle.  ***Post-landing:***  More inversion in CAI (4° from 40-ms to 200-ms post-contact), and up to 3° more DF from 140-ms to 200-ms after the IC.  No statistically significant differences were noted in ankle PF in CAI. | ***Pre-landing:***  More knee ABD in CAI (1.5° from 200-ms pre-IC to IC). More knee flexion in CAI (4.5° from 200-ms to 120-ms pre-IC).  No statistically significant difference was noted in knee ADD in CAI.  ***Post-landing:***  No significant differences in knee sagittal and frontal angles. | ***Pre-landing:***  More hip ABD in CAI (2.5° from 200-ms pre-IC). No statistically significant differences were noted in hip ADD and flexion in CAI.  ***Post-landing:***  No significant differences in hip sagittal and frontal angles. |
| Levin 2015 | Pre-landing (-200ms-0ms)  Post-landing (0ms-300ms)  IC = 0ms for the (potentially) inverting side | NR | NR | NR |
| Li 2018a | Pre-landing (before IC 50ms)  Post-landing (after IC 100ms)  IC = vGRF > 10N | NR | NR | NR |
| Li 2018b | Post-landing phase: from the IC to the first instant when the center of mass reached its lowest height | CAI group displayed 4° less DF and 8° less PF ROM compared with CON.  CAI group had a 5.9° greater inversion angle at IC, and 7.5° less inversion displacement compared to controls.  No statistically significant differences were noted in peak ankle inversion angle after landing. | CAI group exhibited significantly larger values (5.8° difference at IC, 15.7° peak flexion angle, and 8.8° flexion displacement) compared to controls.  No statistically significant differences were noted in peak knee ABD angle. | NR |
| Liu 2013 | Post-landing phase: the time between the foot contact and the maximum knee flexion after the contact. | Greater PF angle on flat surface compared to inverted surface (p=0.023). DF ROM was greater on flat surface than inverted (p=0.001) and combined surface (p<0.001).  The peak inversion in landing on inverted surface was higher than combined surface (p<0.001). The inversion ROMs in landing on inverted surface was greater than combined(p<0.001), and flat surface (p<0.001).  The PF angle was smaller in double-leg landing on flat surface than single-leg landing on flat surface (p=0.017). Greater DF ROM was found in double-leg landing compared to single-leg landing on flat surface (p=0.021).  Peak eversion in double-leg landing on flat surface was smaller than single-leg landing on flat surface (p<0.001). | No significant differences in knee kinematics between healthy and CAI subjects. The knee flexion ROM in landing on combined surface was smaller than that of landing on inverted surface (p=0.015). Knee flexion ROM was greater in double-leg landing on flat surface than single-leg landing on flat surface (p<0.001,).  The knee ADD ROM for healthy subjects was smaller compared to CAI subjects (p=0.003). The knee ADD ROM in landing on flat surface was smaller compared to inverted (p<0.001) and combined (p<0.001) surfaces. | NR |
| Moisan 2020 | Pre-landing phase (from heel off to IC)  Post-landing phase (from the IC to the maximal knee flexion)  IC = vGRF > 10N, the heel lift = foot switch under the heel | Normalized to 0-100% of the landing phase.  *Pre-landing phase:*  NR  *Post-landing:*  For the SIDE, DROP and WEDGE task, no between-group differences were observed for all other muscles and joint angles and moments.  For the FOAM task, the CAI group exhibited increased ankle DF from 73 to 88% (p = 0.02) of the landing phase compared to the control group. No between-group differences were observed for all other muscles and joint angles and moments. | *Pre-landing and Post-landing phase:*  No between group difference was observed for all other muscles and ankle angles and moments. | *Pre-landing and Post-landing phase:*  No between-group differences were observed for all other muscles and joint angles and moments. |
| Simpson 2019 | Pre-landing (before IC 200ms)  Post-landing (after IC 200ms)  IC = vGRF > 15 N | *Pre-landing:*  No significant differences mentioned.  *Post-landing:*  No significant condition by group × interactions for discrete ankle kinematic variables (p > 0.05).  Significant group main effects were observed for time to maximum inversion and maximum inversion angle, with the CAI group demonstrating significantly less time to maximum inversion (p=0.041; d= 0.53) and significantly greater maximum inversion angle (p =0.010; d =0.80).  A significant condition main effect for inversion angle at IC and maximum inversion angle was found, with significant reductions in maximum inversion angle (p < 0.001; d =0.95) and significantly greater inversion angle at IC (p =0.003; d =0.73) during the EXP trial compared to the UE trial.  No statistically significant differences were noted in maximum inversion velocity. | NR | NR |
| Song 2019 | Pre-landing (before IC 200ms)  Post-landing (after IC 200ms)  IC = vGRF ≥ 5% body mass | *Pre-landing:*  The average DF of the injured side in FAI group was significantly greater than that of the healthy side (P < 0.05). No significant differences were found between the injured and healthy sides in other aspects (transverse plane and frontal plane) (P > 0.05). No significant differences were found in joint angles between the FAI group and the control group before landing (P > 0.05).  *Post-landing:*  There was no significant difference between the injured side and the healthy side in the FAI group (P > 0.05). The joint angles of both sides of FAI and control groups are not significantly different (P > 0.05). | NR | NR |
| Watabe 2021a | Pre-landing (IC = vGRF > 10N)  Post-landing (IC = vGRF < 10N) | Each data was normalized to 100% time, with 0% means IC and 100% means toe-off.  *Post-landing:*  The maximum ankle inversion and ankle DF were not significantly different among the CAI and CON groups (p > 0.05). | The maximum knee flexion angles were not significantly different between the CAI and CON groups (p > 0.05). | No significant difference was observed in the maximum hip flexion angle between the CAI and CON groups. |
| Watabe 2021b | Pre-landing (before IC 200ms)  Post-landing (after IC 200ms-400ms)  IC = vGRF＞10N | *Pre-landing:*  No significant difference between groups was observed for the maximum ankle inversion angle during normal landing (p > 0.05).  *Post-landing:*  The maximum ankle inversion angle was significantly increased in the CAI group compared to the control group (p=0.043) during inversion landing.  No significant difference between groups was observed for the ankle inversion angle at IC during both normal and inversion landings (p > 0.05). | NR | NR |
| **Abbreviations:** CAI = chronic ankle instability group; CON = control group; LAS = lateral ankle sprains group; FAI = functional ankle instability group; IC = initial contact; vGRF = vertical component of the ground reaction force; N = newton; SD = standard deviations; NR = not reported; PF = plantarflexion; DF = dorsiflexion; ABD = abduction; ADD = addcution; ROM = range of motion; UE = unexpected; EXP = expected. | | | | |

| **Supporting Information 5 Table S4.** Study outcomes for joint kinetics | | | | |
| --- | --- | --- | --- | --- |
| Study | Outcome Variables | Ankle Kinetics | Knee Kinetics | Hip Kinetics |
| Gutierrez 2012 | Pre-landing (before IC 200ms)  Post-landing (after IC 200ms)  IC = the instant of touchdown = vGRF > 0N (2 SD above baseline noise) | NR | NR | NR |
| Han 2023 | Pre-landing (IC)  Post-landing (after IC 200ms)  IC = vGRF > 15 N | NR | NR | NR |
| Knight 2011 | Latency: the time from contact of the fulcrum with the landing area (initiation of inversion moment) to the time of muscle activity exceeding 5 SD from a baseline muscle activity, which was taken 200 ms before landing.  The time to maximum inversion (ms): the landing area to contact of the lateral border of the outer sole with the landing area. | NR | NR | NR |
| Lee 2023 | Pre-landing (betore IC 200ms)  Post-landing (after IC 200ms)  IC = vGRF > 10N | NR | NR | NR |
| Levin 2015 | Pre-landing (-200ms-0ms)  Post-landing (0ms-300ms)  IC=0ms for the (potentially) inverting side | NR | NR | NR |
| Li 2018a | Pre-landing (before IC 50ms)  Post-landing (after IC 100ms)  IC = vGRF > 10N | NR | NR | NR |
| Li 2018b | Post-landing phase: from the IC to the first instant when the center of mass reached its lowest height | The net joint moments (internal moments) were normalized to participants’ body mass. Eccentric work of the joints was calculated based on integrating the negative part of the power curve and also normalized to body mass.  CAI group had 0.22 Nm/kg lower peak eversion moment, and 0.14 J/kg less frontal plane eccentric work during the landing phase.  No statistically significant differences were noted in ankle peak PF moment and sagittal plane eccentric work. | CAI group exhibited 0.27 Nm/kg peak extension moment, 0.12 Nm/kg peak internal rotation moment, 0.25 J/kg sagittal plane eccentric work) compared to controls.  No statistically significant differences were noted in knee peak ABD moment. | NR |
| Liu 2013 | Landing phase: the time between the foot contact and the maximum knee flexion after the contact. | GRFs were normalized to body weight (BW) and joint moments were normalized to body mass (Nm/kg).  Peak lateral GRF was greater on combined surface compared to flat (p=0.001) and inverted (p=0.002). The time to the peak lateral GRF was shorter in single-leg landing on inverted (p=0.04) and combined (p<0.001) surfaces compared to flat surface, and was also shorter in the combined surface compared to the inverted surface (p<0.001).  Peak vertical GRF was smaller on inverted surface compared to flat (p<0.001) and combined surface (p=0.005). Loading rate of vertical GRF was greater on combined surface compared to flat (p<0.001) and inverted surface (p<0.001), and greater on inverted surface compared to flat surface (p=0.026).  The peak medial GRF in double-leg landing on flat surface was different from the peak lateral GRF in single-leg landing on flat surface (p<0.001).  Peak vertical GRF (p<0.001) and its loading rate (p<0.001) in double-leg landing on flat surface were smaller than flat surface.  The peak PF moment was greater on flat and inverted surfaces compared to combined surface (p<0.001). Smaller peak eversion moment on flat surface compared to inverted (p<0.001) and combined (p<0.001) surfaces. Greater peak PF moment was found in single-leg landing on flat surface than double-leg landing on flat surface (p<0.001). | The knee extension moment in landing on combined surface was greater than flat (p=0.03) and inverted (p<0.001) surfaces. The knee ABD moment in landing on flat surface was smaller than inverted (p<0.001) and combined surface (p<0.001). Peak extension moment (p<0.001) and peak ABD moment (p<0.001) in double-leg landing on flat surface was smaller than single-leg landing on flat surface (p<0.001). | NR |
| Moisan 2020 | Pre-landing phase (from heel off to IC)  Post-landing phase (from the IC to the maximal knee flexion)  IC = vGRF > 10N, the heel lift = foot switch under the heel | Joint angles and moments were normalized to 0 to 100% of the landing phase (from the IC to the maximal knee flexion).  No between-group difference was observed for all other muscles and joint angles and moments. | *Pre-landing phase:*  No between-group difference was observed for all other muscles and joint angles and moments.  *Post-landing phase:*  Only for the WEDGE task, knee extension moment was increased at 22% (p = 0.03) of the landing phase for the CAI compared to the control group. | *Pre-landing phase:*  No between-group difference was observed for all other muscles and joint angles and moments.  *Post-landing phase:*  No between-group difference was observed for all other muscles and joint angles and moments. |
| Simpson 2019 | Pre-landing (before IC 200ms)  Post-landing (after IC 200ms)  IC = vGRF > 15 N | NR | NR | NR |
| Song 2019 | Pre-landing (before IC 200ms)  Post-landing (after IC 200ms)  IC = vGRF ≥ 5% body mass | NR | NR | NR |
| Watabe 2021a | Pre-landing (IC= vGRF > 10N)  Post-landing (IC= vGRF < 10N) | NR | NR | NR |
| Watabe 2021b | Pre-landing (before IC 200ms)  Post-landing (after IC 200ms-400ms)  IC = vGRF > 10N | NR | NR | NR |
| **Abbreviations:** CAI = chronic ankle instability group; CON = control group; IC = initial contact; vGRF = vertical component of the ground reaction force; N = newton; SD = standard deviations; NR = not reported; PF = plantarflexion; ABD = abduction; WEDGE = task of jumping onto a laterally 25° inclined surface. | | | | |

| **Supporting Information 6 Table S5.** Inclusion criteria of studies included in the systematic review based on the recommendation of IAC | | | | | | | | |
| --- | --- | --- | --- | --- | --- | --- | --- | --- |
| **First Author (Year)** | **Population defined by authors** | **All criteria required.** | | | | **At least one of following symptoms.** | | |
|  |  | **A history of at least 1 significant ankle sprain** | **Injury resulting in pain, swelling, interruption of physical activity for at least 1 day** | **The initial ankle sprain occurred at least 12 months prior to the study** | **No history of ankle sprain in last 3 months** | **At least 2 episodes of “giving way” in the last 6 months.** | **Ankle sprain recurrence (at least 2 sprains on same ankle)** | **Self-reported ankle instability confirmed by questionnaires (i.e. at least 5 "yes^†^" in AII, or lower than 24 in CAIT, or larger than 11 in IdFAI)** |
| Gutierrez 2012 | AI, CON | Yes | Unclear | Unclear | Yes | Unclear | No | Yes |
| Han 2023 | CAI, CON | Yes | Yes | Yes | Yes | Yes | Unclear | Yes |
| Knight 2011 | LAS, CON | Yes | Yes | Unclear | Yes | Unclear | Unclear | Yes |
| Lee 2023 | CAI, CON | Yes | Yes | Yes | No | Yes | Unclear | Yes |
| Levin 2015 | CAI, CON | Yes | Yes | Yes | Yes | Yes | Unclear | Yes |
| Li 2018a | CAI, CON | Yes | Yes | Yes | Yes | Yes | Unclear | Yes |
| Li 2018b | CAI, CON | Yes | Yes | Yes | Yes | Yes | Unclear | Yes |
| Liu 2013 | CAI, CON | Yes | Yes | Unclear | Yes | Unclear | No | Yes |
| Moisan 2020 | CAI, CON | Yes | Yes | Yes | Yes | Yes | Unclear | Yes |
| Simpson 2019 | CAI, CON | Yes | Yes | Unclear | Yes | Unclear | Unclear | Yes |
| Song 2018 | FAI, CON | Yes | No | Unclear | Unclear | No | Unclear | Yes |
| Watabe 2021a | CAI, CON | Yes | Yes | Unclear | Yes | Unclear | Unclear | Yes |
| Watabe 2021b | CAI, CON | Yes | Yes | Unclear | Yes | Unclear | Unclear | Yes |
| ^†^Yes = Reported and achieved the standard, No = Not Reported, Unclear = Reported but the details did not achieve the standard.  **Abbreviations:** IAC = International Ankle Consortium; CAI = chronic ankle instability; AI = ankle instability; LAS = lateral ankle sprain; FAI = functional ankle instability; AII = Ankle Instability Instrument; CAIT = Cumberland Ankle Instability Tool; IdFAI = Identification of functional ankle instability. | | | | | | | | |

| **Supporting Information 7 Table S6.** Summary of pooled evidence—electromyography | | | | |
| --- | --- | --- | --- | --- |
| Variable | Comparison | Level of evidence | SMD (95% CI) | Clinical significance |
| PL latency | group | < 0.001* 40% | 1.35 [0.90, 1.80] | A large effect for delayed PL activation after landing in CAI compared with controls. |
|  | UE | < 0.001* 0% | 1.68 [1.11, 2.24] | A large effect for delayed PL activation after landing in CAI compared with controls during unexpected task. |
|  | EXP | 0.13 49% | 0.40 [-0.12, 0.91] |  |
| PB latency | group | 0.68 40% | 0.09 [-0.33, 0.51] | No between-group difference. |
|  | UE | 0.62 | -0.18 [0.90, 0.54] |  |
|  | EXP | 0.39 60% | 0.22 [-0.29, 0.74] |  |
| TA1 | group | 0.03* 41% | 0.28 [0.03, 0.54] | A small effect for higher TA activation before landing in CAI compared with controls. |
|  | UE | 0.17 65% | 0.25 [-0.10, 0.60] |  |
|  | EXP | 0.09 0% | 0.32 [-0.05, 0.70] |  |
| TA2 | group | 0.99 80% | 0.01 [-0.62, 0.64] | No between-group difference. |
|  | UE | 0.30 84% | 0.20 [-0.18, 0.59] |  |
|  | EXP | 0.18 81% | 0.26 [-0.12, 0.65] |  |
| PL1 | group | 0.36 75% | 0.21 [-0.25, 0.68] | No between-group difference. |
|  | UE | 0.12 74% | 0.25 [-0.07, 0.58] |  |
|  | EXP | 0.06 74% | -0.29 [-0.60, 0.02] |  |
| PL2 | group | 0.08 80% | -0.59 [-1.25, 0.07] | No between-group difference. |
|  | UE | 0.18 88% | -0.73 [-1.78, 0.33] |  |
|  | EXP | 0.15 22% | -0.41 [-0.95, 0.14] |  |
| GAS1 | group | 0.22 0% | 0.17 [-0.10, 0.45] | No between-group difference. |
|  | UE | 0.09 0% | 0.30 [-0.05, 0.64] |  |
|  | EXP | 0.83 0% | -0.05 [-0.52, 0.42] |  |
| GAS2 | group | 0.20 73% | -0.39 [-0.99, 0.21] | No between-group difference. |
|  | UE | 0.24 81% | -0.23 [-0.61, 0.15] |  |
|  | EXP | 0.38 68% | -0.22 [-0.70, 0.27] |  |
| fCCI1 | group | 0.08 0% | -0.36 [-0.76, 0.04] | No between-group difference. |
|  | UE | 0.23 0% | -0.29 [-0.76, 0.18] |  |
|  | EXP | 0.46 84% | 0.18 [-0.30, 0.65] |  |
| fCCI2 | group | 0.30 82% | 0.47 [-0.42, 1.35] | No between-group difference. |
|  | UE | 0.05 89% | 0.56 [-0.01, 1.14] |  |
|  | EXP | 0.28 86% | 0.26 [-0.22, 0.74] |  |
| sCCI1 | group | 0.09 24% | 0.29 [-0.05, 0.62] |  |
|  | UE | 0.03* 29% | 0.53 [0.04, 1.01] | A medium effect for higher sagittal co-activation before landing in CAI compared with controls during unexpected task. |
|  | EXP | 0.76 0% | 0.07 [-0.39, 0.54] |  |
| sCCI2 | group | 0.02* 0% | 0.41 [0.06, 0.77] | A small effect for higher sagittal co-activation after landing in CAI compared with controls. |
|  | UE | 0.44 8% | 0.21 [-0.33, 0.75] |  |
|  | EXP | 0.02* 0% | 0.57 [0.09, 1.04] | A medium effect for higher sagittal co-activation after landing in CAI compared with controls during expected task. |
| **Abbreviations:** SMD = standardized mean difference; CI = confidence interval; CAI = chronic ankle instability; UE = unexpected task; EXP = expected task; PL = peroneus longus; PB = peroneus brevis; TA1 = tibialis anterior muscle activity in pre-landing; TA2 = tibialis anterior muscle activity in post-landing; PL1 = peroneus longus muscle activity in pre-landing; PL2 = peroneus longus muscle activity in post-landing; GAS1 = gastrocnemius muscle activity in pre-landing; GAS2 = gastrocnemius muscle activity in post-landing; fCCI1 = frontal plane co-contraction index in pre-landing; fCCI2 = frontal plane co-contraction index in post-landing; sCCI1 = sagittal plane co-contraction index in pre-landing; sCCI2 = sagittal plane co-contraction index in post-landing.  * *P* ≤ 0.05. | | | | |

**Supporting Information 8 Figure S1.** The funnel plots of muscle activity, kinematics and kinetics


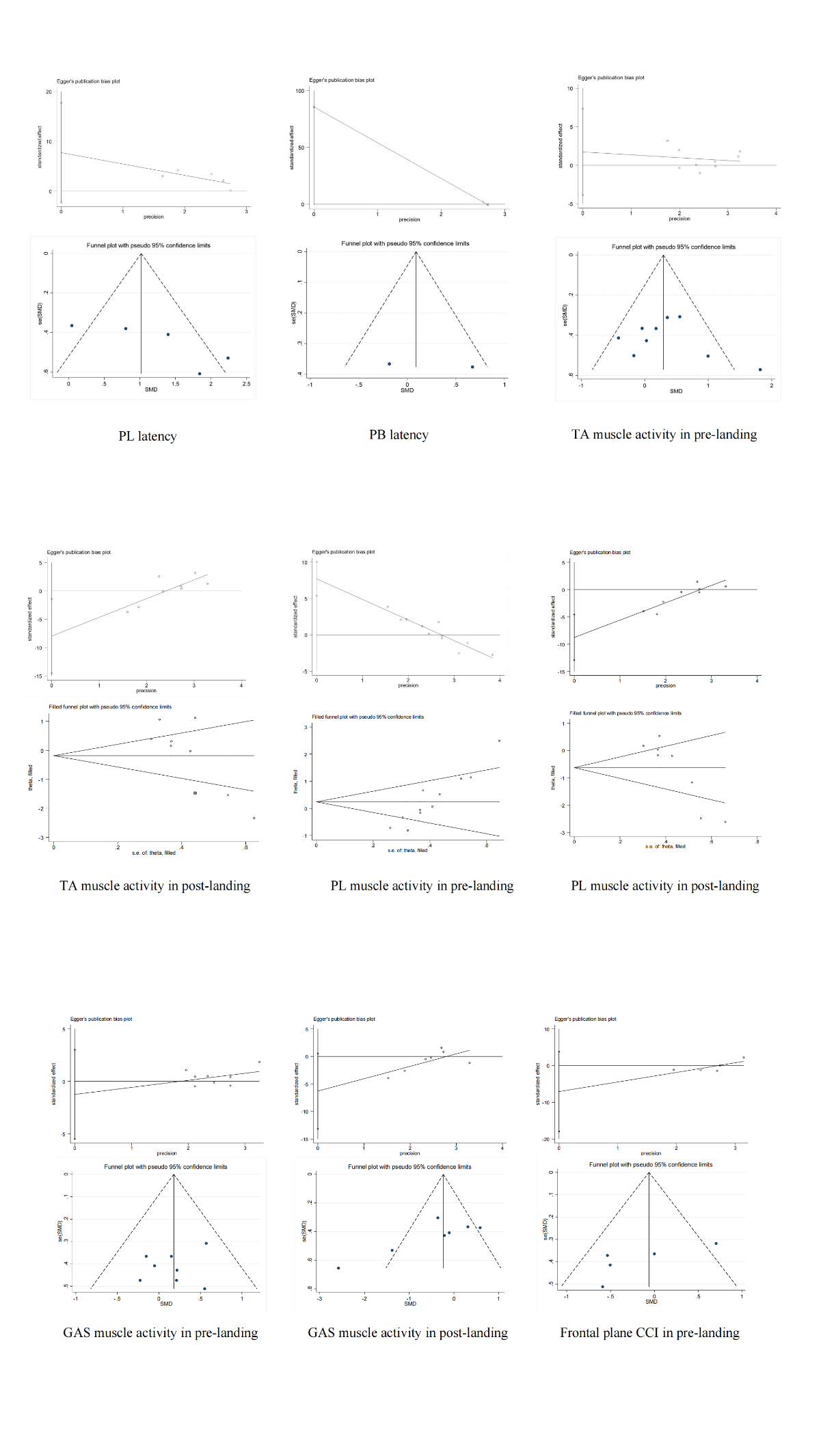


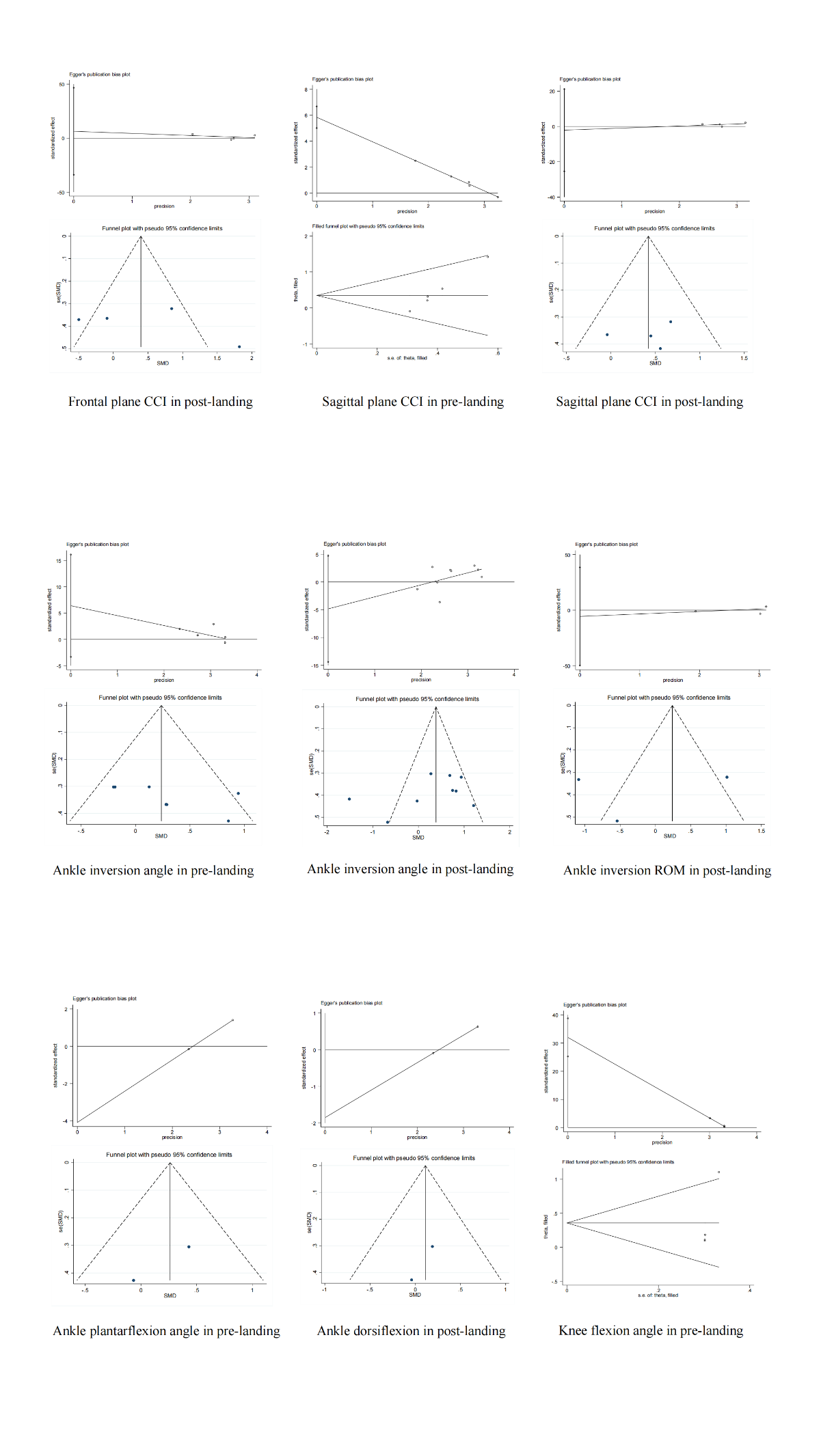


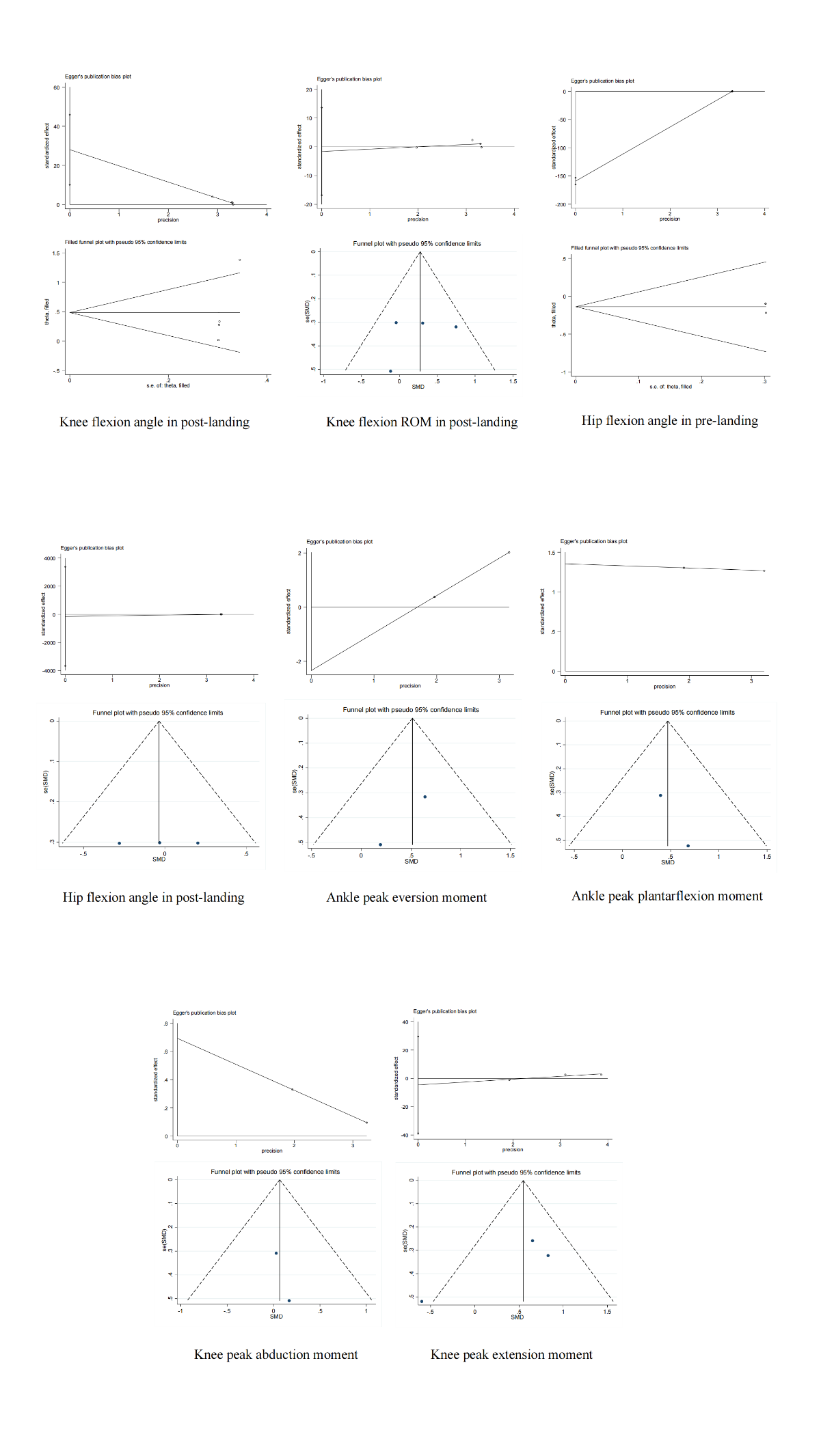


| **Supporting Information 9 Table S7.** Results of sensitivity analyses | | | | |
| --- | --- | --- | --- | --- |
| Analysis | SMD | 95% CI | *P*-value | *I*^2^ |
| *PL latency* | | | | |
| Main analysis | 0.98 | 0.60, 1.36 | < 0.001* | 72% |
| Influential cases removed^1†^ | 1.35 | 0.90, 1.80 | < 0.001* | 40% |
| *Frontal plane CCI in pre-landing* | | | | |
| Main analysis | -0.12 | -0.64, 0.39 | 0.64 | 56% |
| Influential cases removed^2^ | -0.36 | -0.76, 0.04 | 0.08 | 0% |
| *Ankle inversion angle in post-landing* | | | | |
| Main analysis | \| 0.38 \| \| --- \| | 0.13, 0.62 | 0.002* | 76% |
| Influential cases removed^3^ | \| 0.56 \| \| --- \| | 0.30, 0.81 | < 0.001* | 41% |
| *Knee flexion angle in pre-landing* | | | | |
| Main analysis | \| \| 0.33 \| \| --- \| \| \| --- \| --- \| | 0.03, 0.63 | 0.03* | 54% |
| Influential cases removed^4^ | \| 0.13 \| \| --- \| | -0.22, 0.47 | 0.47 | 0% |
| *Knee flexion angle in post-landing* | | | | |
| Main analysis | \| \| 0.44 \| \| --- \| \| \| --- \| --- \| | 0.13, 0.74 | 0.005* | 68% |
| Influential cases removed^5^ | \| 0.20 \| \| --- \| | -0.14, 0.55 | 0.25 | 0% |
| *Ankle inversion ROM in post-landing* | | | | |
| Main analysis | \| \| 0.30 \| \| --- \| \| \| --- \| --- \| | -0.05, 0.64 | 0.09 | 89% |
| Influential cases removed^6^ | \| 0.83 \| \| --- \| | 0.42, 1.24 | < 0.001* | 38% |
| *Knee peak extension moment* | | | | |
| Main analysis | \| \| 0.54 \| \| --- \| \| \| --- \| --- \| | 0.17, 0.91 | 0.004* | 63% |
| Influential cases removed^7^ | \| 0.71 \| \| --- \| | 0.32, 1.11 | < 0.001* | 0% |
| ^†^Removed as influential cases: ^1^Knight 2011, ^2^Li 2018a, ^3^Gutierrez 2012, ^4^Li 2018b, ^5^Li 2018b, ^6^Li 2018b, ^7^Liu 2013.  **Abbreviations:** SMD = standardized mean difference; CI = confidence interval.  * *P* ≤ 0.05. | | | | |

| **Supporting Information 10 Table S8.** Summary of pooled evidence—kinematics and kinetics | | | | | |
| --- | --- | --- | --- | --- | --- |
| Variable | Comparison | Level of evidence | SMD (95% CI) | MD (95% CI)^†^ | Clinical significance |
| AI1 | group | 0.07 43% | 0.23 [-0.02, 0.48] | 1.07° [0.16°, 1.97°] |  |
|  | UE | 0.69 39% | 0.07 [-0.27, 0.40] |  |  |
|  | EXP | 0.02* 43% | 0.43 [0.06, 0.81] |  | A small effect for higher ankle inversion angle before landing in CAI compared with controls during expected task. |
| AI2 | group | < 0.001* 41% | 0.56 [0.30, 0.81] | 2.41° [1.44°, 3.39°] | A medium effect for higher ankle inversion angle after landing in CAI compared with controls. |
|  | UE | 0.002* 85% | 0.50 [0.18, 0.82] |  | A medium effect for higher ankle inversion angle after landing in CAI compared with controls during unexpected task. |
|  | EXP | 0.29 38% | 0.20 [-0.17, 0.58] |  |  |
| AIROM2 | group | < 0.001* 38% | 0.83 [0.42, 1.24] | 4.25° [2.42°, 6.07°] | A large effect for higher ankle inversion displacement in CAI compared with controls. |
|  | UE | 0.002* | 1.00 [0.37, 1.62] |  |  |
|  | EXP | 0.90 90% | -0.03 [-0.44, 0.39] |  |  |
| APF1 | group | 0.30 0% | 0.26 [-0.23, 0.74] | 1.67° [-2.31°, 5.65°] | No between-group difference. |
| ADF2 | group | 0.65 0% | 0.11 [-0.37, 0.59] | 0.62° [-3.00°, 4.24°] | No between-group difference. |
| AEM | group | 0.06 0% | 0.50 [-0.02, 1.03] |  | No between-group difference. |
| APFM | group | 0.09 0% | 0.46 [-0.07, 0.98] |  | No between-group difference. |
| KF1 | group | 0.47 0% | 0.13 [-0.22, 0.47] | 0.52° [-0.98°, 2.02°] |  |
|  | UE | 0.51 0% | 0.14 [-0.28, 0.56] |  |  |
|  | EXP | 0.02* 79% | 0.54 [0.10, 0.98] |  | A medium effect for higher knee flexion angle before landing in CAI compared with controls during expected task. |
| KF2 | group | 0.25 0% | 0.20 [-0.14, 0.55] | 1.39° [-1.28°, 4.06°] |  |
|  | UE | 0.50 0% | 0.14 [-0.28, 0.56] |  |  |
|  | EXP | < 0.001* 80% | 0.77 [0.33, 1.22] |  | A medium effect for higher knee flexion angle after landing in CAI compared with controls during expected task. |
| KFROM2 | group | 0.11 20% | 0.27 [-0.06, 0.60] | 1.88° [-0.80°, 4.56°] |  |
|  | UE | 0.89 | -0.04 [-0.63, 0.55] |  |  |
|  | EXP | 0.04* 9% | 0.41 [0.01, 0.81] |  | A small effect for higher knee flexion displacement in CAI compared with controls during expected task. |
| KABM | group | 0.81 0% | 0.06 [-0.45, 0.58] |  | No between-group difference. |
| KEM | group | < 0.001* 0% | 0.71 [0.32, 1.11] |  | A medium effect for higher knee extension moment in CAI compared with controls. |
| HF1 | group | 0.43 0% | -0.14 [-0.48, 0.21] | -1.07° [-3.65°, 1.50°] | No between-group difference. |
|  | UE | 0.46 0% | -0.16 [-0.58, 0.26] |  |  |
|  | EXP | 0.75 | -0.10 [-0.69, 0.49] |  |  |
| HF2 | group | 0.84 0% | -0.03 [-0.38, 0.31] | -0.34° [-3.40°, 2.73°] | No between-group difference. |
|  | UE | 0.48 0% | -0.15 [-0.57, 0.27] |  |  |
|  | EXP | 0.51 | 0.20 [-0.39, 0.79] |  |  |
| ^†^ MD was calculated by the data of main analyses.  **Abbreviations:** SMD = standardized mean difference; MD = mean difference; CI = confidence interval; CAI = chronic ankle instability; UE = unexpected task; EXP = expected task; AI1 = ankle inversion angle in pre-landing; AI2 = ankle inversion angle in post-landing; AIROM2 = ankle inversion range of motion in post-landing; APF1 = ankle plantarflexion angle in pre-landing; ADF2 = ankle dorsiflexion angle in post-landing; AEM = ankle peak eversion moment; APFM = ankle peak plantarflexion moment; KF1 = knee flexion angle in pre-landing; KF2 = knee flexion angle in post-landing; KFROM2 = knee flexion range of motion in post-landing; KABM = knee peak abduction moment; KEM = knee peak extension moment; HF1 = hip flexion angle in pre-landing; HF2 = hip flexion angle in post-landing.  * *P* ≤ 0.05. | | | | | |

| **Supporting Information 11 Table S9.** The results from the changes in heterogeneity in each subgroup analysis | | | | | | |
| --- | --- | --- | --- | --- | --- | --- |
| Outcome | Number of studies | Number of participants | Heterogeneity | | Meta analysis | |
|  |  |  | *P* value | I^2^ | Effect estimate (95%) | *P* value |
| PL latency | 5 | 130 | 0.007* | 72% | 0.98 [0.60, 1.36] | < 0.001* |
| UE | 3 | 70 | 0.49 | 0% | 1.68 [1.11, 2.24] | < 0.001* |
| EXP | 2 | 60 | 0.16 | 49% | 0.40 [-0.12, 0.91] | 0.13 |
| PB latency | 3 | 90 | 0.19 | 40% | 0.09 [-0.33, 0.51] | 0.37 |
| UE | 1 | 30 |  |  | -0.18 [0.90, 0.54] | 0.62 |
| EXP | 2 | 60 | 0.11 | 60% | 0.22 [-0.29, 0.74] | 0.39 |
| TA1 | 9 | 244 | 0.09 | 41% | 0.28 [0.03, 0.54] | 0.03* |
| UE | 5 | 132 | 0.02* | 65% | 0.25 [-0.10, 0.60] | 0.17 |
| EXP | 4 | 112 | 0.53 | 0% | 0.32 [-0.05, 0.70] | 0.09 |
| TA2 | 8 | 228 | < 0.001* | 80% | 0.23 [-0.04, 0.51] | 0.10 |
| UE | 4 | 116 | < 0.001* | 84% | 0.20 [-0.18, 0.59] | 0.30 |
| EXP | 4 | 112 | 0.001* | 81% | 0.26 [-0.12, 0.65] | 0.18 |
| PL1 | 11 | 337 | < 0.001* | 75% | -0.03 [-0.25, 0.19] | 0.78 |
| UE | 6 | 162 | 0.002* | 74% | 0.25 [-0.07, 0.58] | 0.12 |
| EXP | 5 | 175 | 0.004* | 74% | -0.29 [-0.60, 0.02] | 0.06 |
| PL2 | 8 | 216 | < 0.001* | 80% | -0.59 [-1.25, -0.07] | 0.08 |
| UE | 5 | 146 | < 0.001* | 88% | -0.73 [-1.78, 0.33] | 0.18 |
| EXP | 3 | 70 | 0.28 | 22% | -0.41 [-0.95, 0.14] | 0.15 |
| GAS1 | 8 | 202 | 0.80 | 0% | 0.17 [-0.10, 0.45] | 0.22 |
| UE | 5 | 132 | 0.76 | 0% | 0.30 [-0.05, 0.64] | 0.09 |
| EXP | 3 | 70 | 0.75 | 0% | -0.05 [-0.52, 0.42] | 0.83 |
| GAS2 | 7 | 186 | 0.001* | 73% | -0.22 [-0.52, 0.08] | 0.14 |
| UE | 4 | 116 | 0.001* | 81% | -0.23 [-0.61, 0.15] | 0.24 |
| EXP | 3 | 70 | 0.04* | 68% | -0.22 [-0.70, 0.27] | 0.38 |
| fCCI1 | 5 | 142 | 0.06 | 56% | -0.06 [-0.39, 0.28] | 0.74 |
| UE | 3 | 70 | 0.57 | 0% | -0.29 [-0.76, 0.18] | 0.23 |
| EXP | 2 | 72 | 0.01* | 84% | 0.18 [-0.30, 0.65] | 0.46 |
| fCCI2 | 4 | 126 | < 0.001* | 82% | 0.39 [0.02, 0.75] | 0.04* |
| UE | 2 | 54 | 0.003* | 89% | 0.56 [-0.01, 1.14] | 0.05 |
| EXP | 2 | 72 | 0.008* | 86% | 0.26 [-0.22, 0.74] | 0.28 |
| sCCI1 | 5 | 142 | 0.26 | 24% | 0.29 [-0.05, 0.62] | 0.09 |
| UE | 3 | 70 | 0.24 | 29% | 0.53 [0.04, 1.01] | 0.03* |
| EXP | 2 | 72 | 0.41 | 0% | 0.07 [-0.39, 0.54] | 0.76 |
| sCCI2 | 4 | 126 | 0.52 | 0% | 0.41 [0.06, 0.77] | 0.02* |
| UE | 2 | 54 | 0.30 | 8% | 0.21 [-0.33, 0.75] | 0.44 |
| EXP | 2 | 72 | 0.65 | 0% | 0.57 [0.09, 1.04] | 0.02* |
| AI1 | 7 | 258 | 0.10 | 43% | 0.23 [-0.02, 0.48] | 0.07 |
| UE | 4 | 142 | 0.18 | 39% | 0.07 [-0.27, 0.40] | 0.69 |
| EXP | 3 | 116 | 0.17 | 43% | 0.43 [0.06, 0.81] | 0.02* |
| AI2 | 9 | 285 | < 0.001* | 76% | 0.38 [0.13, 0.62] | 0.002* |
| UE | 5 | 172 | < 0.001* | 85% | 0.50 [0.18, 0.82] | 0.002* |
| EXP | 4 | 113 | 0.18 | 38% | 0.20 [-0.17, 0.58] | 0.29 |
| AIROM2 | 4 | 147 | < 0.001* | 89% | 0.28 [-0.07, 0.62] | 0.11 |
| UE | 1 | 44 |  |  | 1.00 [0.37, 1.62] | 0.002* |
| EXP | 3 | 103 | 0.317 | 90% | -0.03 [-0.44, 0.39] | 0.90 |
| KF1 | 4 | 174 | 0.09 | 54% | 0.33 [0.03, 0.63] | 0.03* |
| UE | 2 | 88 | 0.86 | 0% | 0.14 [-0.28, 0.56] | 0.51 |
| EXP | 2 | 86 | 0.02* | 79% | 0.54 [0.10, 0.98] | 0.02* |
| KF2 | 4 | 174 | 0.02* | 68% | 0.44 [0.13, 0.74] | 0.005* |
| UE | 2 | 88 | 0.55 | 0% | 0.14 [-0.28, 0.56] | 0.50 |
| EXP | 2 | 86 | 0.03* | 80% | 0.77 [0.33, 1.22] | < 0.001* |
| KFROM2 | 4 | 147 | 0.29 | 20% | 0.27 [-0.06, 0.60] | 0.11 |
| UE | 1 | 44 |  |  | -0.04 [-0.63, 0.55] | 0.89 |
| EXP | 3 | 103 | 0.33 | 9% | 0.41 [0.01, 0.81] | 0.04* |
| HF1 | 3 | 132 | 0.95 | 0% | -0.14 [-0.48, 0.21] | 0.43 |
| UE | 2 | 88 | 0.78 | 0% | -0.16 [-0.58, 0.26] | 0.46 |
| EXP | 1 | 44 |  |  | -0.10 [-0.69, 0.49] | 0.75 |
| HF2 | 3 | 132 | 0.54 | 0% | -0.03 [-0.38, 0.31] | 0.84 |
| UE | 2 | 88 | 0.57 | 0% | -0.15 [-0.57, 0.27] | 0.48 |
| EXP | 1 | 44 |  |  | 0.20 [-0.39, 0.79] | 0.51 |
| **Abbreviations:** CI = confidence interval; PL = peroneus longus; PB = peroneus brevis; TA1 = tibialis anterior muscle activity in pre-landing; TA2 = tibialis anterior muscle activity in post-landing; PL1 = peroneus longus muscle activity in pre-landing; PL2 = peroneus longus muscle activity in post-landing; GAS1 = gastrocnemius muscle activity in pre-landing; GAS2 = gastrocnemius muscle activity in post-landing; fCCI1 = frontal plane co-contraction index in pre-landing; fCCI2 = frontal plane co-contraction index in post-landing; sCCI1 = sagittal plane co-contraction index in pre-landing; sCCI2 = sagittal plane co-contraction index in post-landing; AI1 = ankle inversion angle in pre-landing; AI2 = ankle inversion angle in post-landing; AIROM2 = ankle inversion range of motion in post-landing; KF1 = knee flexion angle in pre-landing; KF2 = knee flexion angle in post-landing; KFROM2 = knee flexion range of motion in post-landing; HF1 = hip flexion angle in pre-landing; HF2 = hip flexion angle in post-landing.  * *P* ≤ 0.05. | | | | | | |
